# Comparative Genomic Analysis of Soil Dwelling Bacteria Utilizing a Combinational Codon Usage and Molecular Phylogenetic Approach Accentuating on Key Housekeeping Genes

**DOI:** 10.3389/fmicb.2019.02896

**Published:** 2019-12-17

**Authors:** Jayanti Saha, Barnan K. Saha, Monalisha Pal Sarkar, Vivek Roy, Parimal Mandal, Ayon Pal

**Affiliations:** ^1^Microbiology & Computational Biology Laboratory, Department of Botany, Raiganj University, Raiganj, India; ^2^Mycology & Plant Pathology Laboratory, Department of Botany, Raiganj University, Raiganj, India

**Keywords:** codon usage bias (CUB), molecular phylogenetics, soil bacteria, housekeeping genes, *atpD* gene, *infB* gene, *trpB* gene, *rpoB* gene

## Abstract

Soil is a diversified and complex ecological niche, home to a myriad of microorganisms particularly bacteria. The physico-chemical complexities of soil results in a plethora of physiological variations to exist within the different types of soil dwelling bacteria, giving rise to a wide variation in genome structure and complexity. This serves as an attractive proposition to analyze and compare the genome of a large number soil bacteria to comprehend their genome complexity and evolution. In this study a combination of codon usage and molecular phylogenetics of the whole genome and key housekeeping genes like *infB* (translation initiation factor 2), *trpB* (tryptophan synthase, beta subunit), *atpD* (ATP synthase, beta subunit), and *rpoB* (RNA polymerase, beta subunit) of 92 soil bacterial species spread across the entire eubacterial domain and residing in different soil types was performed. The results indicated the direct relationship of genome size with codon bias and coding frequency in the studied bacteria. The codon usage profile demonstrated by the gene *trpB* was found to be relatively different from the rest of the housekeeping genes with a large number of bacteria having a greater percentage of genes with Nc values less than the Nc of *trpB*. The results from the overall codon usage bias profile also depicted that the codon usage bias in the key housekeeping genes of soil bacteria was majorly due to selectional pressure and not mutation. The analysis of hydrophobicity of the gene product encoded by the *rpoB* coding sequences demonstrated tight clustering across all the soil bacteria suggesting conservation of protein structure for maintenance of form and function. The phylogenetic affinities inferred using 16S rRNA gene and the housekeeping genes demonstrated conflicting signals with *trpB* gene being the noisiest one. The housekeeping gene *atpD* was found to depict the least amount of evolutionary change in the soil bacteria considered in this study except in two *Clostridium* species. The phylogenetic and codon usage analysis of the soil bacteria consistently demonstrated the relatedness of *Azotobacter chroococcum* with different species of the genus *Pseudomonas*.

## Introduction

Soil, from the biological point of view, can be considered as one of the great ecological component or a system where a vast majority of microorganisms exists, albeit unexplored (Daniel, 2005; Stefanis et al., 2013; Jansson and Hofmockel, 2018). Soil hosts a wide range of microbial community that has a profound effect on the properties of soil itself (Handelsman et al., 1998; Aislabie et al., 2013), and exerts a great influence on the organism's survival and gradual evolution while bestowing unique biological properties (Delgado and Gómez, 2016).

Soil has always been an enigma, a *terra incognita* with diversified habitat and diverse array of interactions (Whitman et al., 1998; Huang et al., 2001; Fierer et al., 2014; Andújar et al., 2017) but scientific understanding of the microbial world in soil is somewhat poor, particularly that of soil bacteria (Fierer and Jackson, 2006; Nesme et al., 2016; Jansson and Hofmockel, 2018). There exists a great variability in the microbial population of soil and the adeptness to explore the soil microbial communities could provide answers related to different functional and phylogenetic aspects of soil microorganisms (Nesme et al., 2016). In general, soil biomass is occupied by about 70% microorganisms which helps in the decomposition of the soil organic matter and releasing the essential minerals on the soil surface (Hayat et al., 2010; Jacoby et al., 2017). Microbes from three different domains of life are found in soil (Rao, 1995), and the largest one to dominate the soil habitat are members of the domain Eubacteria. Each of the different types of bacteria residing in the soil owns distinct morphological, physiological, biochemical, and ecological characteristics, and the variation in the structure and composition of different soil types impart a great influence on the diversity of the microbial community that the soil retain (Fierer and Jackson, 2006; Berg and Smalla, 2009). Hence, microbes from different soil habitat must possess a variation in the genome structure and function (Martin-Laurent et al., 2001; Roesch et al., 2007) to cope with this variability. That's why the ancestry and evolution of the microbial population residing in different types of soil needs to be better understood (Mocali and Benedetti, 2010). Many studies relating to soil microbial ecology has already revealed the immense diversity of soil bacteria, and now it is a very common hypothesis that plant species as well as the soil type have a substantial influence on the structure and function of the rhizosphere associated bacterial populations (Berg and Smalla, 2009; Pérez-Valera et al., 2015). Some of the major soil inhabiting bacterial genera which are of great interest include different species of the genera *Bacillus, Clostridium, Pseudomonas, Streptomyces, Micromonospora, Nitrosomonas, Nitrobacter, Vibrio*, and *Thiobacillus* (Shimp et al., 1993; Lehman et al., 2015).

Among the different types of microbes studied, soil dwelling bacteria offers a lot of promise but a lot of it still remains unexplored. A considerable large scale and robust comparative genomic and phylogenetic analysis of such bacteria is still deficient. Now-a-days the accessibility of the complete genome sequences of many soil borne bacteria provides a scope to undertake various bioinformatics based approaches (Chen et al., 2002; Mardis, 2008). With the rapid and continuous advancements in sequencing technology and data analysis, the complexity of the microbial world is becoming understandable, albeit slowly (Winsley et al., 2012). The recent widespread availability and affordability of automated sequencing has gained much more attention for cataloging soil bacterial diversity (Will et al., 2010) in the era of Next Generation Sequencing (Clooney et al., 2016). Simultaneously the dramatic progress in computing power provides greater opportunities for a large scale and robust *in silico* based study. The acquisition of large amount of sequence data from genes as well as proteins from several repositories (Kaminuma et al., 2010; Chen et al., 2017) is paving the way for comparative genomics in a diverse group of microorganisms (Rosselli et al., 2016) whose major objective is to decipher the genetic variation among different species, and determine the genetic relatedness between closely related organisms. This paves the way to reveal the evolutionary relationship between organisms with the help of conserved sequences that are generally found within the last common ancestors of those organisms (Ivanova et al., 2003; Herring et al., 2006).

Phylogenetic inference using sequence data is considered an important and reliable tool (Ludwig and Schleifer, 1994). To focus on bacterial phylogeny, ribosomal RNA is often envisaged as one of the best parameter due to its ubiquitous and informative nature (Morales et al., 2009) but it's not without its pitfalls. Phylogenetic analysis based on the 16S rRNA gene is generally used to articulate the evolutionary history (Rajendhran and Gunasekaran, 2011) but, several discrepancies have been observed in 16S rRNA based phylogeny (Vásquez-Ponce et al., 2018). Some of the major drawbacks include mosaicism due to horizontal gene transfer and recombination events (Eardly et al., 1996; Schouls et al., 2003), presence of polymorphic genes (Janda and Abbott, 2007; Lang et al., 2013), intra-genomic heterogeneity of the 16S rRNA genes, presence of multiple copies of the rRNA operon (Wang et al., 1997; Klappenbach et al., 2000), etc. To overcome these, the housekeeping genes are now considered to be better molecular markers and as a result these have been widely applied to infer proper phylogenetic relationships (Lai et al., 2014; Tyler et al., 2018). Due to the evolutionary conserved nature, some housekeeping genes like *atpD, infB, gyrB, rpoB, trpB* have been increasingly utilized to improve the discriminatory power in determining the phylogenetic relationships (Martens et al., 2008). Each and every house keeping gene possess the functional constancy and conservation as they encode core metabolic enzymes, and are more or less universally present in a plethora of organisms. An excellent feature demonstrated by the housekeeping genes is the power to overcome conflicting signals from horizontal gene transfer and recombination during phylogeny inference (Case et al., 2007). Therefore, it is a very resourceful proposition to compare the sequences of several housekeeping genes as well as the 16S rRNA genes portraying as a reference for evaluating a comprehensive bacterial phylogeny, and also for investigating the genetic as well as physiological relatedness within the species. A technique which combines analysis of several housekeeping genes called multilocus sequence analysis (MLSA) has emerged as a powerful and pragmatic molecular method to assess pertinent phylogenetic information in the field of polyphasic taxonomy (Das et al., 2014; Vásquez-Ponce et al., 2018). Several studies have been carried out using MLSA in order to get better resolution regarding the phylogenetic analysis and inter-species relatedness in a host of soil bacterial genera like *Arthrobacter* (Liu et al., 2018)*, Borrelia* (Margos et al., 2009)*, Burkholderia* (Estrada-De Los Santos et al., 2013), *Ensifer* (Martens et al., 2008)*, Micromonospora* (Carro et al., 2012), *Streptomyces* (Rong and Huang, 2010), *Nocardia* (Barcellos et al., 2007; McTaggart et al., 2010; Serrano et al., 2010; Busse and Wieser, 2014), and some other rhizospheric bacteria like *Mesorhizobium* (Degefu et al., 2011) and *Bradyrhizobium* (Delamuta et al., 2012).

In addition to molecular phylogenetics, codon usage bias (CUB) within and across the genome also stands as an increasingly demanding factor contributing significantly to gene and genome evolution. CUB is the phenomenon of usage of certain specific codons more frequently than other synonymous codons (Quax et al., 2015), and universally affects the genome of all living beings (Yannai et al., 2018). This is observed mainly due to the degenerate property of the genetic code (Salim and Cavalcanti, 2008; Sharp et al., 2010). It represents balance between translational selection and mutational forces leading to translational efficiency of genes (Sharp et al., 2010; Plotkin and Kudla, 2011). Evidences suggest that strong CUB is generally observed in highly expressed genes (Bennetzen and Hall, 1982; Gouy and Gautier, 1982; Hershberg and Petrov, 2008; Salim and Cavalcanti, 2008; Frumkin et al., 2018), and it is also a means to fine tune the expression of genes (Quax et al., 2015; Sahoo et al., 2019).

Soil is a crucial but diversified habitat, and a wide variety of bacteria lives in soil, demonstrating massive variation in terms of morphology, physiology and biochemistry. Depending on the soil composition and physical attributes the biotic components of soil are going to be diverse but, there is a possibility that every soil dwelling bacteria might have a unifying signature within their genome indicating their origin and evolution in the soil habitat. In this study, we have made an extensive comparative codon usage and phylogenetic analysis of 92 soil dwelling bacterial species distributed within 36 genera spanning the entire eubacterial domain. These 92 bacterial species are unified by the fact that they dwell in soil, albeit of diverse types. The key objectives of this study was to find out if there exists any signature codon usage profile within the genome and major housekeeping genes of soil bacteria. The housekeeping genes are part of core or minimal set of genes primarily responsible for maintaining critical cellular functions (Wei et al., 2018), mainly located on chromosomes with orthologs in related species (Bittner et al., 2007; Villaseñor et al., 2011). The four key housekeeping genes considered in this study are *atpD, infB, rpoB*, and *trpB*. These genes play a significant role in genetic information processing and metabolic activities of bacteria (Wen et al., 2016). The *atpD* gene encodes the beta subunit of ATP synthase that produces ATP from ADP in the presence of a proton gradient across the membrane, whereas, the *infB* gene encodes the translation initiation factor II, which is essential for prokaryotic protein synthesis initiation. The *rpoB* gene encodes the highly conserved portion of the bacterial beta subunit in DNA-dependent RNA polymerase enzyme whereas the *trpB* gene encodes the beta subunit of tryptophan synthase enzyme. In this study, we have also tried to deduce the phylogenetic affinities of the 92 bacterial species considered in this study both at the organismal as well as gene level. We have tried to capture whether the organismal phylogeny based on 16S rRNA gene is corroborated by the individual housekeeping genes or there lies any conflicting signal between them. In addition to this, we have also tried to find out whether the phylogenetic affinities demonstrated by the individual housekeeping genes are in sync with each other. In order to achieve this we have deliberately avoided the use of concatenated housekeeping gene sequences in constructing phylogenies (Thiergart et al., 2014), which have been a popular choice in constructing bacterial phylogeny (Baldauf et al., 2000; Brown et al., 2001; Wu and Eisen, 2008; Tambong et al., 2014). Studies utilizing codon usage parameters like codon adaptation index have been used in the past to detect environmental signatures in bacteria (Willenbrock et al., 2006) but, our study is the first one of its kind where we have adopted a bipartite combinational codon usage and phylogenetic analysis of whole genome as well as key housekeeping genes. This was done to obtain a better resolution into the intraspecific diversity and evolutionary relationships among the different soil dwelling bacteria.

## Materials and Methods

### Whole Genome Sequence Retrieval

The whole genome sequence of 92 soil bacteria were obtained from Integrated Microbial Genome Database (Markowitz et al., 2012) and NCBI genome database (Benson et al., 2006). Selection of these bacterial species were carried out after thoroughly confirming their soil dwelling nature in consultation with available literature including Bergey's Manual of Systematic Bacteriology (Garrity et al., 2001; Brenner et al., 2005; Vos et al., 2009; Krieg et al., 2010; Whitman et al., 2012), and the metadata associated with the respective genome data. Genomes of bacterial species with improper metadata regarding habitat and source of isolation, and incomplete annotation of the coding sequences were deliberately kept out of the analysis. Draft genomes of bacterial species were also not considered for the analysis. Species without strain demarcation were also left out since ‘strain' forms an integral component of the bacterial species concept (Rosselló-Mora and Amann, 2001). The selected 92 bacterial species considered in this study is spread across the entire Eubacterial domain belonging to the five different taxonomic classes (namely Proteobacteria, Firmicutes, Chlorobi, Actinobacteria, and Acidobacteria) spread over 20 different orders and 27 different families. A list of these organisms is given in Supplementary Table 1. Genes with correct initiation and termination codons were considered for every genome to minimize sampling error.

### 16S rRNA Gene and Housekeeping Gene Sequences Retrieval

The nucleotide sequences of 16S rRNA gene and that of the housekeeping genes *atpD, infB, rpoB*, and *trpB* of the 92 bacterial genera were mined from the whole genome sequences to study codon usage profile and construct multiple sequence alignment (MSA) to infer phylogenetic relationships.

### Analysis of CUB

The parameters like effective number of codons or Nc (Wright, 1990), GC content at the third position of the codon or GC3 (Wright, 1990), hydrophobicity and gene length was calculated. Nc is one of the best and widely used measure that quantifies the extent to which the usage of a gene departs from the equal usage of synonymous codons (Fuglsang, 2006; Liu, 2013). It ranges from 20 to 61. A Nc value lower than 40 indicates codon usage bias and higher than 40 indicates equal likely usage of all synonymous codons (Botzman and Margalit, 2011; Pal et al., 2015; Wang et al., 2018; Khandia et al., 2019). GC3 has been reported to be linked with DNA flexibility and codon bias across prokaryotes (Babbitt et al., 2014) and also plays a regulatory role in gene expression and methylation (Tatarinova et al., 2013). In the present study, GC3 was calculated for both the whole genome and every housekeeping gene. Hydrophobicity is actually used for the prediction of the nature of cellular protein coded by the corresponding gene present within the genome. A value below zero indicates the presence of hydrophilic protein and above zero represents the presence of hydrophobic protein (Magdeldin et al., 2012). In our study, we have used this parameter to estimate the physical attribute hydrophobicity of the housekeeping gene products coded by *rpoB, atpD, infB*, and *trpB* gene. The Nc-plot depicting the correlation between Nc and GC3 (Wright, 1990) was constructed to determine the variation in inter-specific as well as inter genic synonymous codon usage pattern existing within the whole genome and the housekeeping genes of the soil bacteria. Nc plot is used extensively to elucidate the mechanistic forces shaping CUB, and heterogeneity of the gene using codon bias and base composition (Sun et al., 2016; Wang et al., 2018; Khandia et al., 2019). In this study, the above mentioned parameters were estimated for the whole genomes and housekeeping genes using INCA (Supek and Vlahovicek, 2004) and CodonW (Peden, 1999).

### Generation of Multiple Sequence Alignments (MSAs)

MSAs of the 16S rRNA genes and the four housekeeping genes from the 92 bacterial species were prepared using the web interface of Clustal Omega hosted at the EMBL-EBI website (https://www.ebi.ac.uk/Tools/msa/clustalo/). Clustal Omega is a MSA tool that produces alignments between multiple sequences employing seeded guide trees and HMM profile-profile techniques (Sievers et al., 2011).

### Phylogenetic Analysis

Phylogenetic inferences using the 16S rRNA gene and the four housekeeping genes were deduced using the maximum likelihood (ML) method. In terms of accuracy, time, and convenience, the ML method is regarded as one of the most appropriate tree estimating method providing information regarding evolutionary relationships (Mulet et al., 2010). Besides, this method utilizes likelihood function and can be used easily to detect the pair wise distances in the phylograms (Rong and Huang, 2014). Since evolutionary models (or models of nucleotide substitution) is a key component of molecular phylogenetics using methods, such as ML, for each gene, the optimal model of evolution was selected using the model test function of MEGA 6.0 (Kumar et al., 2008). The Bayesian Information Criterion (BIC) value has been regarded as an essential criteria for model selection and the models depicting the lowest BIC value were selected as optimal models (Posada and Buckley, 2004; Posada, 2009; Luo et al., 2010). The phylogenetic trees were constructed using MEGA 6.0 (Kumar et al., 2008). For the four housekeeping genes considered in this study, the General Time Reversible model (Nei and Kumar, 2000) with gamma (G) distributed rate variation among sites along with significant proportion of invariable sites (I) or GTR + G + I model was found to be the optimal one based on the BIC score. The 16S rRNA gene based phylogenetic tree was inferred based on the Kimura 2-parameter model (Kimura, 1980). The bootstrap consensus tree inferred from 1,000 replicates (Felsenstein, 1985) was taken to represent the evolutionary history of the analyzed taxa. The visualization and annotation of the phylogenetic trees were done using the online open source tool Interactive Tree of Life (iTOL) ver. 4.4.2 available at https://itol.embl.de/ (Letunic and Bork, 2007, 2019). The raw data containing the phylogenetic trees along with bootstrap support is provided as Supplementary Files in Newick format.

## Results and Discussion

In this study, the codon usage profile of the whole genome along with the housekeeping gene of 92 soil dwelling bacterial species belonging to 36 different genera were thoroughly analyzed. Simultaneously, molecular phylogenetic analysis was carried out by comparing the standard 16S rRNA gene based tree in the backdrop with the phylograms obtained using four key housekeeping genes. A detailed list of the 92 selected bacterial species is given in Supplementary Table 1.

### Analysis of CUB Pattern of Whole Genome

CUB analysis is a fitting proposition for understanding the functional evolution of genome within and between species. The CUB phenomenon has been found to control a wide range of cellular processes, such as translational efficiency, differential protein production and its subsequent folding (Quax et al., 2015; Song et al., 2017a; Zhang et al., 2018), and is being influenced by a plethora of factors but not restricted to GC content, gene length, gene hydropathy, gene function and expression, protein structure, mutational bias and compositional bias (Tuller et al., 2010; Plotkin and Kudla, 2011; Xu et al., 2011, 2013; Chithambaram et al., 2014). In this study, the primary codon usage parameters like Nc, GC3, gene length and hydrophobicity of 391,415 gene sequences, representing the whole genome, of 92 soil dwelling bacterial species were estimated. It was found that the average genomic Nc (Nc_avg_) of all the studied taxa lies between 29 and 54. The highest Nc_avg_ value of 53.58 with a standard deviation (SD) of ±4.32 was depicted by *Nitrosomonas communis* Nm2, a Gram negative Mediterranean soil dwelling organism. About 52 species from different soil types were found to demonstrate Nc_avg_ above 40. This is suggestive of a relatively lower codon bias existing within the genome of these species (Botzman and Margalit, 2011; Pal et al., 2015; Khandia et al., 2019). The lowest Nc_avg_ value (29.99, *SD* = ±3.91) was observed in *Micrococcus luteus* NCTC 2665, a Gram positive soil bacterium indicating high level of codon bias within its genome. Other organisms that demonstrated higher genomic codon usage bias includes species of *Micromonospora, Clostridium, Streptomyces, Achromobacter, Acidiphilium, Nocardia*, etc. The standard deviation within the genomic Nc of each organism was also estimated. The lowest deviation of Nc within the genome was observed in *Clostridium butyricum* JKY6D1 (*SD* = ±3.34), a Gram positive bacteria isolated from pit mud whereas the highest deviation in Nc was observed in *Azotobacter chroococcum* NCIMB 8003 (*SD* = ±7.17), a Gram negative soil bacterium. The results of the whole genome CUB analysis is given in Table 1.

**Table 1:** List of genomic Nc (Nc_avg_) and genomic GC3 (GC3_avg_) along with standard deviation in the 92 species of soil bacteria analyzed in this study.

**Organism name**	**Genomic Nc (Nc_**avg**_)**	**Standard deviation of Nc_**avg**_**	**Genomic GC3 (GC3_**avg**_)**	**Standard deviation of GC3_**avg**_**
*Acidocella aminolytica* DSM 11237	46.19	6.19	0.74	0.1
*Acidobacterium capsulatum* ATCC 51196	41.72	7.09	0.8	0.1
*Acidiphilium cryptum* JF-5	35.58	5.62	0.91	0.08
*Actinoalloteichus cyanogriseus* DSM 43889	32.94	3.46	0.91	0.05
*Acidovorax delafieldii* 2AN	37.3	6.82	0.85	0.091
*Achromobacter denitrificans* NBRC 15125	32.9	4.39	0.94	0.054
*Acidithiobacillus ferrivorans* SS3	49.1	5.73	0.7	0.108
*Acidithiobacillus ferrooxidans* ATCC 23270	47.23	5.83	0.75	0.098
*Acinetobacter calcoaceticus* PHEA-2	45.43	5.05	0.3	0.08
*Acidithiobacillus caldus* SM-1	45.34	6.13	0.77	0.092
*Acidiphilium multivorum* AIU301	37.17	6.68	0.89	0.1
*Acidithiobacillus thiooxidans* ATCC 19377	49.53	5.1	0.65	0.098
*Achromobacter xylosoxidans* A8	35.29	5.87	0.9	0.072
*Agrobacterium tumefaciens* 5A	44.88	5.8	0.75	0.072
*Alcaligenes faecalis* P156	44.47	5.15	0.71	0.08
*Azotobacter chroococcum* NCIMB 8003	34.37	7.17	0.89	0.102
*Bacillus akibai* JCM 9157	47.55	4.76	0.27	0.072
*Bacillus atrophaeus* 1942	52.53	4.92	0.49	0.115
*Bacillus azotoformans* LMG 9581	49.48	5.2	0.31	0.082
*Bacillus circulans* NBRC 13626	46.97	4.92	0.29	0.077
*Bacillus clausii* KSM-K16	53.14	4.4	0.48	0.084
*Bacillus cohnii* NBRC 15565	46.17	4.9	0.26	0.074
*Bacillus drentensis* NBRC 102427	51.82	5.22	0.37	0.097
*Bacillus firmus* NBRC 15306	52.36	4.88	0.43	0.1
*Bacillus flexus* Riq5	48.77	5.19	0.32	0.082
*Bacillus horikoshii* DSM 8719	53	5.25	0.4	0.101
*Bacillus krulwichiae* NBRC 102362	48.32	4.86	0.29	0.08
*Bacillus megaterium* WSH-002	48.95	5.13	0.32	0.08
*Bacillus methanolicus* MGA3	51.19	4.92	0.36	0.08
*Bacillus niacini* NBRC 15566	50.99	4.92	0.33	0.08
*Bacillus novalis* NBRC 102450	52.62	5.13	0.41	0.11
*Bacillus pseudofirmus* OF4	50.57	5.14	0.34	0.08
*Bacillus pseudomycoides* DSM 12442	44.4	5.19	0.25	0.07
*Bacillus pumilus* NJ-V2	51.81	5.07	0.4	0.09
*Bacillus simplex* SH-B26	52.98	5.44	0.4	0.1
*Bacillus soli* NBRC 102451	52.52	5.07	0.4	0.1
*Bacillus vallismortis* DV1-F-3	52.65	4.84	0.49	0.12
*Bacillus vireti* LMG 21834	52.62	4.97	0.41	0.11
*Bdellovibrio bacteriovorus* HD100	47.5	4.98	0.62	0.11
*Beggiatoa alba* B18LD	46.09	3.99	0.44	0.09
*Beijerinckia indica indica* ATCC 9039	46.69	5.54	0.74	0.09
*Brevibacillus agri* BAB-2500	46.81	5.74	0.71	0.13
*Burkholderia ambifaria* IOP40-10	34.79	6.23	0.9	0.08
*Burkholderia anthina* AZ-4-2-10-S1-D7	33.65	5.27	0.92	0.07
*Chlorobium phaeovibrioides* DSM 265	49.15	4.75	0.6	0.08
*Chromobacterium subtsugae* MWU2387	34.18	6.51	0.9	0.11
*Chromobacterium vaccinii* 21-1	34.29	6.16	0.9	0.09
*Clostridium acetobutylicum* EA 2018	39.63	3.87	0.15	0.05
*Clostridium argentinense* CDC 2741	37.22	4.12	0.13	0.06
*Clostridium butyricum* JKY6D1	35.93	3.34	0.1	0.05
*Clostridium cadaveris* NLAE-zl-G419	38.64	4.05	0.13	0.06
*Clostridium cochlearium* NLAE-zl-C224	35.46	3.89	0.12	0.05
*Clostridium pasteurianum* DSM 525 = ATCC 6013	38.86	4.27	0.17	0.06
*Clostridium scatologenes* ATCC 25775	37.26	4.01	0.12	0.06
*Clostridium sporogenes* NCIMB 10696	35.92	4.2	0.13	0.05
*Clostridium tetani* 12124569	36.22	3.75	0.13	0.05
*Desulfobacterium autotrophicum* HRM2, DSM 3382	50.5	4.43	0.59	0.1
*Desulfobacter postgatei* 2ac9	51.08	4.72	0.59	0.11
*Desulfocapsa sulfexigens* DSM 10523	53.33	3.97	0.46	0.08
*Desulfobacula toluolica* Tol2	51.36	4.06	0.47	0.1
*Flavobacterium pectinovorum* DSM 6368	44.65	5.05	0.26	0.09
*Flavobacterium suncheonense* GH29-5, DSM 17707	48.92	5.39	0.49	0.15
*Hyphomicrobium denitrificans* 1NES1	45.9	5.75	0.74	0.07
*Micromonospora aurantiaca* ATCC 27029	30.94	3.68	0.93	0.05
*Micromonospora carbonacea* DSM 43168	30.37	3.84	0.95	0.06
*Micromonospora chokoriensis* DSM 45160	32.76	3.68	0.9	0.05
*Micromonospora echinospora* DSM 43816	31.66	3.79	0.92	0.05
*Micrococcus luteus* NCTC 2665	29.99	3.91	0.95	0.05
*Micromonospora purpureochromogenes* DSM 43821	30.68	3.93	0.94	0.05
*Nitrosomonas communis* Nm2	53.58	4.32	0.47	0.11
*Nitrosomonas europaea* ATCC 19718	51.29	4.55	0.58	0.1
*Nitrobacter hamburgensis* X14	42.46	6.34	0.81	0.09
*Nitrobacter winogradskyi* Nb-255	42.18	5.47	0.82	0.08
*Nocardia cerradoensis* NBRC 101014	36.42	4.14	0.88	0.05
*Nocardia otitidiscaviarum* IFM 11049	35.12	4.34	0.89	0.05
*Pseudomonas azotoformans* S4	40.16	6.64	0.81	0.1
*Pseudomonas citronellolis* P3B5	30.22	6.2	0.93	0.09
*Pseudomonas fluorescens* A506	40.55	6.41	0.8	0.1
*Pseudomonas mendocina* NK-01	37.69	6.15	0.81	0.08
*Pseudomonas oryzihabitans* USDA-ARS-USMARC-56511	35.46	4.96	0.87	0.07
*Pseudomonas putida* 1A00316	34.37	6.43	0.87	0.09
*Rhizobium gallicum* IE4872	44.3	6.45	0.76	0.08
*Streptomyces avermitilis* MA-4680	33.27	4.15	0.91	0.06
*Streptomyces clavuligerus* ATCC 27064	32.89	4.08	0.92	0.06
*Streptomyces hygroscopicus limoneus* KCTC 1717	31.82	4.24	0.92	0.06
*Streptomyces noursei* ATCC 11455	32.47	4.94	0.92	0.07
*Streptomyces rubidus* CGMCC 4.2026	31.06	3.98	0.94	0.05
*Streptomyces scabrisporus* DSM 41855	33.76	3.92	0.9	0.05
*Streptomyces vitaminophilus* ATCC 31673	32.37	3.56	0.92	0.05
*Thiobacillus denitrificans* ATCC 25259	36.2	5.29	0.89	0.07
*Vibrio gazogenes* DSM 21264	52.1	4.53	0.5	0.11
*Vibrio natriegens* NBRC 15636	51.65	6.35	0.42	0.1

The average genomic GC3 content (GC3_avg_) of the organisms under study was found to range between 10 and 95%. *Clostridium butyricum* JKY6D1 with a GC3_avg_ value of 10% demonstrated the lowest average genomic GC3 content. On the other hand, *Micrococcus luteus* NCTC 2665 and *Micromonospora carbonacea* DSM 43168 depicted the highest GC3_avg_ score of 95%. Both these organisms are Gram positive in nature.

In this study, we have tried to determine if the Nc_avg_ is related to the genome size (measured in terms of base count), coding frequency and GC content of the genome (data shown in Supplementary Table 2). To explore this relationship Spearman's rank order correlation was performed. Our results demonstrated that the Nc_avg_ is significantly negatively correlated with genome size (ρ = −0.442, *p* < 0.01), coding bases (ρ = −0.46, *p* < 0.01) and GC content (ρ = −0.577, *p* < 0.01), respectively in the soil bacteria. Alternatively, it implies that the genomic codon usage bias bears a positive correlation with the genome size. We also observed that in these 92 bacterial species there is a significant positive correlation between the genome size and the coding frequency of the genome (ρ = 0.992, *p* < 0.01). This simultaneously suggests that for these species, there is no significant superfluity of nucleotides in the genome, since majority are engaged in coding function. Coding frequency was also found to correlate significantly in a positive manner with the GC content of the genome (ρ = 0.84, *p* < 0.01). The increasing GC content within the genome is thus suggestive of better genomic coding efficiency and increased codon bias within these 92 bacterial species. Our results thus reflect a unified trend in the soil dwelling species where genome size is found to be proportional to the codon bias and the coding frequency.

In most of the species a significant negative correlation between Nc and GC3 was depicted, a trend well in line with the standard notion of Nc-GC3 relationship (Hassan et al., 2009; Pandit and Sinha, 2011; Prabha et al., 2012; Malakar et al., 2016, 2019; Song et al., 2017b). The results of the correlation analysis is shown in Supplementary Table 3. Out of the 92 species, 51 were found to depict such a trend, whereas 32 species exhibited substantial positive correlation between Nc and GC3. An interesting observation was the lack of substantial relationship between Nc and GC3 in the two organisms *Bdellovibrio bacteriovorus* HD100 and *Nitrosomonas communis* Nm2. Both these bacteria are terrestrial Gram negative soil dwellers, aerobic, free living and isolated from Mediterranean soil. The dispersion within the GC3 of the 92 organisms showed that the GC3 value deviated from 5 to 15% within the 92 organisms with most of the organisms depicting about 5% deviation. The highest deviation in GC3 was demonstrated by *Flavobacterium suncheonense* GH29-5, DSM 17707 (15%) which is a Gram negative, greenhouse soil living bacterium. But, *F. pectinovorum* DSM 6368 though a member of the genus *Flavobacterium* demonstrated only 9% deviation in its GC3 content. Genera like *Clostridium, Pseudomonas, Micromonospora, Bacillus*, and *Streptomyces* exhibited similar trend.

### Comparative CUB Profile Analysis of Key Housekeeping Genes in Soil Bacteria

To overcome several discrepancies and conflicting signals like mosaicism due to horizontal gene transfer, instances of recombination and presence of polymorphic genes in 16S rRNA based phylogenetic analysis, several housekeeping genes are considered as potent tool in determining bacterial taxonomy (Soler et al., 2004). Each and every housekeeping gene possess the functional constancy and conservation as they encode core metabolic enzymes and are generally present in all common members (Naser et al., 2005). In this study, the housekeeping genes *atpD, infB, rpoB*, and *trpB* have been utilized to improve the discriminatory power in determining the phylogenetic relationships and resolve the phylogenetic discrepancies cropping out while using 16SrRNA genes. All these housekeeping genes are closely involved with the core metabolic pathways and genetic information processing pathways found in bacteria. The findings of this study have been detailed in the succeeding section.

### Comparative CUB Analysis of *rpoB* Gene From Soil Bacteria

**Figure 1:**
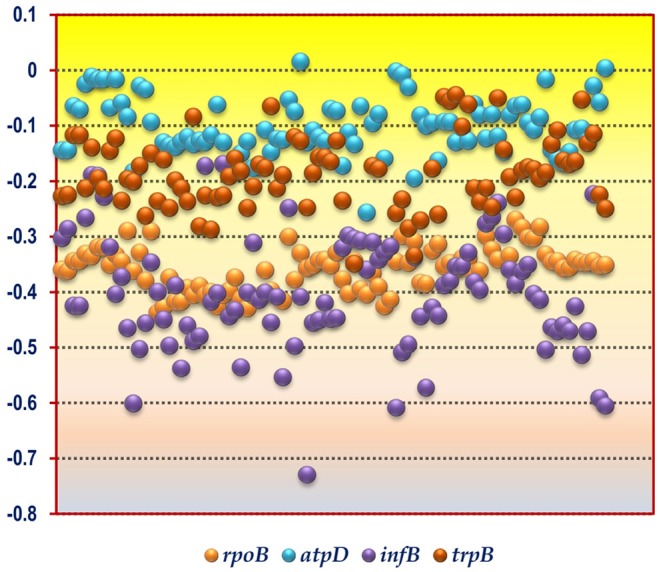
A scattered plot depicting the hydrophobicity profile of the gene products encoded by the four housekeeping genes *rpoB, atpD, infB*, and *trpB* from the soil bacterial species considered in this study. The *y*-axis corresponds to the hydrophobicity value whereas the *x*-axis corresponds to the bacterial species sorted in alphabetical order as given in Table 1.

The CUB profile of *rpoB* gene was found to reflect the genomic CUB profile. The lowest Nc value of *rpoB* (Nc = 26) was depicted by the organism *Micrococcus luteus* NCTC 2665 which also depicted the lowest Nc_avg_ of 29.99. A similar trend was also observed in the genus *Nitrosomonas* which exhibited the highest genic Nc value for *rpoB* (*N. europaea* ATCC 19718, Nc = 51.58) and genome (*N. communis* Nm2, Nc_avg_ = 53.58). The lowest GC3 content for *rpoB* was demonstrated by the genus *Clostridium* which also had the lowest genomic GC3 content. The organism *Micrococcus luteus* NCTC 2665 did not show the highest GC3 value for *rpoB* although it possessed a significantly high mean genomic GC3 content (96.33%). The *rpoB* of *Acidiphilium multivorum* was found to possess the highest GC3 content (97%) which is somewhat incongruent with respect to its genomic GC3 pattern. The CUB profile of *rpoB* have been illustrated in Tables 2, 3. In case of hydrophobicity of *rpoB* gene product, the lowest hydrophobicity (−0.437) was reported by *Bacillus akibai* JCM 9157 (Gram positive, free living soil bacteria) while the Gram negative Pseudomonads like *Pseudomonas citronellolis* P3B5, *P. putida* 1A00316, and *P. fluorescens* A506 depicted the maximum hydrophobicity ranging from −0.269 to −0.284. A scattered plot showing the distribution of hydrophobicity values of the protein encoded by *rpoB* gene is given in Figure 1.

**Table 2:** Nc profile of the four housekeeping genes in 92 soil bacterial species.

**Organism name**	***rpoB* Nc**	**Nc_**avg**_-*rpoB* Nc**	***atpD* Nc**	**Nc_**avg**_-*atpD* Nc**	***infB* Nc**	**Nc_**avg**_-*infB* Nc**	***trpB* Nc**	**Nc_**avg**_-*trpB* Nc**
*Acidocella aminolytica* DSM 11237	37.50	8.69	34.19	12.00	37.28	8.91	38.91	7.28
*Acidobacterium capsulatum* ATCC 51196	33.24	8.48	33.32	8.40	38.09	3.63	37.54	4.18
*Acidiphilium cryptum* JF-5	29.18	6.40	27.56	8.02	35.89	−0.31	29.49	6.09
*Actinoalloteichus cyanogriseus* DSM 43889	29.64	3.30	28.27	4.67	31.67	1.27	30.44	2.50
*Acidovorax delafieldii* 2AN	32.41	4.89	32.68	4.62	34.50	2.80	32.43	4.87
*Achromobacter denitrificans* NBRC 15125	29.66	3.24	28.49	4.41	29.47	3.43	30.33	2.57
*Acidithiobacillus ferrivorans* SS3	49.59	−0.49	40.55	8.55	49.24	−0.14	56.32	−7.22
*Acidithiobacillus ferrooxidans* ATCC 23270	44.97	2.26	44.24	2.99	45.08	2.15	45.71	1.52
*Acinetobacter calcoaceticus* PHEA-2	37.48	7.95	35.81	9.62	36.15	9.28	38.95	6.48
*Acidithiobacillus caldus* SM-1	39.19	6.15	39.77	5.57	39.83	5.51	45.63	−0.29
*Acidiphilium multivorum* AIU301	28.95	8.22	27.24	9.93	36.35	0.82	29.38	7.79
*Acidithiobacillus thiooxidans* ATCC 19377	46.53	3.00	43.34	6.19	47.62	1.91	46.88	2.65
*Achromobacter xylosoxidans* A8	30.07	5.22	29.60	5.69	29.22	6.07	30.09	5.20
*Agrobacterium tumefaciens* 5A	34.75	10.13	32.46	12.42	37.37	7.51	39.47	5.41
*Alcaligenes faecalis* P156	38.28	6.19	35.55	8.92	40.07	4.40	44.19	0.28
*Azotobacter chroococcum* NCIMB 8003	32.16	2.21	27.19	7.18	33.18	1.19	26.72	7.65
*Bacillus akibai* JCM 9157	40.58	6.97	0.00	47.55	43.52	4.03	49.97	−2.42
*Bacillus atrophaeus* 1942	47.54	4.99	44.16	8.37	49.10	3.43	54.72	−2.19
*Bacillus azotoformans* LMG 9581	46.67	2.81	39.11	10.37	42.59	6.89	46.55	2.93
*Bacillus circulans* NBRC 13626	40.70	6.27	40.87	6.10	40.85	6.12	44.80	2.17
*Bacillus clausii* KSM-K16	51.29	1.85	50.22	2.92	48.68	4.46	53.87	−0.73
*Bacillus cohnii* NBRC 15565	43.14	3.03	37.06	9.11	39.46	6.71	48.39	−2.22
*Bacillus drentensis* NBRC 102427	46.28	5.54	42.63	9.19	48.19	3.63	48.87	2.95
*Bacillus firmus* NBRC 15306	47.66	4.70	42.53	9.83	47.23	5.13	52.57	−0.21
*Bacillus flexus* Riq5	39.18	9.59	36.91	11.86	40.47	8.30	48.72	0.05
*Bacillus horikoshii* DSM 8719	47.47	5.53	37.72	15.28	43.34	9.66	57.20	−4.20
*Bacillus krulwichiae* NBRC 102362	42.97	5.35	40.65	7.67	40.26	8.06	49.34	−1.02
*Bacillus megaterium* WSH-002	38.87	10.08	37.08	11.87	39.92	9.03	46.71	2.24
*Bacillus methanolicus* MGA3	48.89	2.30	44.07	7.12	48.58	2.61	50.84	0.35
*Bacillus niacini* NBRC 15566	43.49	7.50	40.37	10.62	44.14	6.85	52.62	−1.63
*Bacillus novalis* NBRC 102450	48.77	3.85	43.63	8.99	48.95	3.67	51.61	1.01
*Bacillus pseudofirmus* OF4	42.98	7.59	37.15	13.42	44.27	6.30	49.54	1.03
*Bacillus pseudomycoides* DSM 12442	39.35	5.05	35.43	8.97	39.16	5.24	44.55	−0.15
*Bacillus pumilus* NJ-V2	46.80	5.01	40.39	11.42	47.68	4.13	53.81	−2.00
*Bacillus simplex* SH-B26	46.13	6.85	38.89	14.09	45.46	7.52	56.43	−3.45
*Bacillus soli* NBRC 102451	50.58	1.94	44.96	7.56	47.69	4.83	47.03	5.49
*Bacillus vallismortis* DV1-F-3	0.00	52.65	43.92	8.73	50.79	1.86	55.11	−2.46
*Bacillus vireti* LMG 21834	47.94	4.68	44.60	8.02	50.12	2.50	52.54	0.08
*Bdellovibrio bacteriovorus* HD100	38.05	9.45	33.31	14.19	41.15	6.35	0.00	47.50
*Beggiatoa alba* B18LD	48.30	−2.21	40.32	5.77	48.50	−2.41	47.32	−1.23
*Beijerinckia indica indica* ATCC 9039	39.78	6.91	37.56	9.13	42.08	4.61	44.61	2.08
*Brevibacillus agri* BAB-2500	45.03	1.78	39.33	7.48	45.72	1.09	45.06	1.75
*Burkholderia ambifaria* IOP40-10	29.79	5.00	28.80	5.99	28.86	5.93	29.06	5.73
*Burkholderia anthina* AZ-4-2-10-S1-D7	28.67	4.98	28.79	4.86	29.24	4.41	28.95	4.70
*Chlorobium phaeovibrioides* DSM 265	48.43	0.72	47.96	1.19	47.60	1.55	46.72	2.43
*Chromobacterium subtsugae* MWU2387	27.84	6.34	30.31	3.87	28.12	6.06	30.65	3.53
*Chromobacterium vaccinii* 21-1	28.85	5.44	31.12	3.17	30.90	3.39	32.31	1.98
*Clostridium acetobutylicum* EA 2018	37.93	1.70	32.25	7.38	36.29	3.34	36.16	3.47
*Clostridium argentinense* CDC 2741	37.19	0.03	40.54	−3.32	33.63	3.59	0.00	37.22
*Clostridium butyricum* JKY6D1	34.60	1.33	32.30	3.63	32.98	2.95	37.88	−1.95
*Clostridium cadaveris* NLAE-zl-G419	37.91	0.73	32.81	5.83	32.89	5.75	0.00	38.64
*Clostridium cochlearium* NLAE-zl-C224	35.12	0.34	34.29	1.17	33.71	1.75	0.00	35.46
*Clostridium pasteurianum* DSM 525 = ATCC 6013	36.54	2.32	35.42	3.44	34.25	4.61	34.67	4.19
*Clostridium scatologenes* ATCC 25775	34.68	2.58	33.44	3.82	33.13	4.13	33.06	4.20
*Clostridium sporogenes* NCIMB 10696	34.28	1.64	32.60	3.32	33.72	2.20	0.00	35.92
*Clostridium tetani* 12124569	35.06	1.16	0.00	36.22	32.03	4.19	0.00	36.22
*Desulfobacterium autotrophicum* HRM2, DSM 3382	49.66	0.84	46.19	4.31	53.08	−2.58	52.28	−1.78
*Desulfobacter postgatei* 2ac9	50.07	1.01	44.07	7.01	49.09	1.99	40.78	10.30
*Desulfocapsa sulfexigens* DSM 10523	47.97	5.36	55.38	−2.05	48.30	5.03	51.08	2.25
*Desulfobacula toluolica* Tol2	51.15	0.21	38.74	12.62	50.29	1.07	50.63	0.73
*Flavobacterium pectinovorum* DSM 6368	38.61	6.04	36.86	7.79	39.47	5.18	45.79	−1.14
*Flavobacterium suncheonense* GH29-5, DSM 17707	44.04	4.88	41.36	7.56	47.33	1.59	0.00	48.92
*Hyphomicrobium denitrificans* 1NES1	32.11	13.79	32.41	13.49	41.29	4.61	43.07	2.83
*Micromonospora aurantiaca* ATCC 27029	28.58	2.36	27.12	3.82	31.49	−0.55	27.21	3.73
*Micromonospora carbonacea* DSM 43168	28.45	1.92	27.53	2.84	30.84	−0.47	28.37	2.00
*Micromonospora chokoriensis* DSM 45160	30.03	2.73	28.85	3.91	31.03	1.73	28.90	3.86
*Micromonospora echinospora* DSM 43816	28.28	3.38	27.16	4.50	30.71	0.95	27.30	4.36
*Micrococcus luteus* NCTC 2665	26.00	3.99	24.83	5.16	27.74	2.25	28.44	1.55
*Micromonospora purpureochromogenes* DSM 43821	28.94	1.74	27.46	3.22	31.37	−0.69	26.91	3.77
*Nitrosomonas communis* Nm2	49.89	3.69	45.09	8.49	48.37	5.21	56.33	−2.75
*Nitrosomonas europaea* ATCC 19718	51.59	−0.30	50.25	1.04	51.56	−0.27	54.07	−2.78
*Nitrobacter hamburgensis* X14	30.39	12.07	32.25	10.21	31.70	10.76	35.06	7.40
*Nitrobacter winogradskyi* Nb-255	33.13	9.05	32.84	9.34	31.66	10.52	35.01	7.17
*Nocardia cerradoensis* NBRC 101014	32.32	4.10	29.28	7.14	28.07	8.35	34.78	1.64
*Nocardia otitidiscaviarum* IFM 11049	0.00	35.12	28.38	6.74	28.08	7.04	31.71	3.41
*Pseudomonas azotoformans* S4	32.71	7.45	34.20	5.96	36.25	3.91	30.43	9.73
*Pseudomonas citronellolis* P3B5	28.05	2.17	27.58	2.64	29.73	0.49	25.44	4.78
*Pseudomonas fluorescens* A506	34.57	5.98	34.30	6.25	36.57	3.98	31.77	8.78
*Pseudomonas mendocina* NK-01	30.26	7.44	30.27	7.42	31.31	6.38	27.35	10.34
*Pseudomonas oryzihabitans* USDA-ARS-USMARC-56511	32.14	3.32	28.44	7.02	33.78	1.68	29.83	5.63
*Pseudomonas putida* 1A00316	31.75	2.62	32.11	2.26	32.16	2.21	25.44	8.93
*Rhizobium gallicum* IE4872	31.52	12.78	32.43	11.87	37.16	7.14	34.63	9.67
*Streptomyces avermitilis* MA-4680	28.77	4.50	27.90	5.37	30.74	2.53	27.33	5.94
*Streptomyces clavuligerus* ATCC 27064	28.66	4.23	29.79	3.10	29.07	3.82	29.09	3.80
*Streptomyces hygroscopicus limoneus* KCTC 1717	28.64	3.18	27.09	4.73	29.33	2.49	27.47	4.35
*Streptomyces noursei* ATCC 11455	28.33	4.14	28.36	4.11	29.46	3.01	26.90	5.57
*Streptomyces rubidus* CGMCC 4.2026	27.22	3.84	26.76	4.30	29.34	1.72	26.76	4.30
*Streptomyces scabrisporus* DSM 41855	28.95	4.81	30.20	3.56	30.55	3.21	28.55	5.21
*Streptomyces vitaminophilus* ATCC 31673	28.18	4.19	29.01	3.36	31.01	1.36	28.32	4.05
*Thiobacillus denitrificans* ATCC 25259	31.69	4.51	29.17	7.03	31.76	4.44	31.79	4.41
*Vibrio gazogenes* DSM 21264	44.23	7.87	40.85	11.25	44.28	7.82	51.70	0.40
*Vibrio natriegens* NBRC 15636	35.91	15.74	54.74	−3.09	37.30	14.35	51.51	0.14

*Nc_avg_-rpoB Nc, Nc_avg_-atpD Nc, Nc_avg_-infB Nc, and Nc_avg_-trpB Nc denotes the difference in Nc values between the genomic Nc and the corresponding housekeeping gene Nc*.

**Table 3:** GC3 profile of the four housekeeping genes in 92 soil bacterial species.

**Organism name**	***rpoB* GC3**	**GC3_**avg**_-rpoB GC3**	***atpD* GC3**	**GC3_**avg**_-*atpD* GC3**	***infB* GC3**	**GC3_**avg**_-*infB* GC3**	***trpB* GC3**	**GC3_**avg**_-*trpB* GC3**
*Acidocella aminolytica* DSM 11237	0.78	−0.04	0.76	−0.02	0.81	−0.07	0.86	−0.12
*Acidobacterium capsulatum* ATCC 51196	0.88	−0.08	0.86	−0.06	0.81	−0.01	0.87	−0.07
*Acidiphilium cryptum* JF-5	0.96	−0.05	0.97	−0.06	0.88	0.03	1.00	−0.09
*Actinoalloteichus cyanogriseus* DSM 43889	0.93	−0.02	0.92	−0.01	0.83	0.08	0.92	−0.01
*Acidovorax delafieldii* 2AN	0.84	0.01	0.82	0.03	0.82	0.03	0.89	−0.04
*Achromobacter denitrificans* NBRC 15125	0.88	0.06	0.87	0.07	0.92	0.02	0.95	−0.01
*Acidithiobacillus ferrivorans* SS3	0.66	0.04	0.79	−0.09	0.67	0.03	0.55	0.15
*Acidithiobacillus ferrooxidans* ATCC 23270	0.73	0.02	0.78	−0.03	0.74	0.01	0.79	−0.04
*Acinetobacter calcoaceticus* PHEA-2	0.17	0.13	0.16	0.14	0.15	0.15	0.25	0.05
*Acidithiobacillus caldus* SM-1	0.83	−0.06	0.75	0.02	0.83	−0.06	0.79	−0.02
*Acidiphilium multivorum* AIU301	0.97	−0.08	0.96	−0.07	0.86	0.03	0.99	−0.10
*Acidithiobacillus thiooxidans* ATCC 19377	0.66	−0.01	0.67	−0.02	0.61	0.04	0.71	−0.06
*Achromobacter xylosoxidans* A8	0.86	0.04	0.86	0.04	0.88	0.02	0.93	−0.03
*Agrobacterium tumefaciens* 5A	0.73	0.02	0.67	0.08	0.70	0.05	0.71	0.04
*Alcaligenes faecalis* P156	0.63	0.08	0.57	0.14	0.63	0.08	0.67	0.04
*Azotobacter chroococcum* NCIMB 8003	0.86	0.03	0.87	0.02	0.82	0.07	0.96	−0.07
*Bacillus akibai* JCM 9157	0.20	0.07	0.00	0.27	0.22	0.05	0.31	−0.04
*Bacillus atrophaeus* 1942	0.31	0.18	0.32	0.17	0.41	0.08	0.44	0.05
*Bacillus azotoformans* LMG 9581	0.25	0.06	0.13	0.18	0.21	0.10	0.28	0.03
*Bacillus circulans* NBRC 13626	0.16	0.13	0.14	0.15	0.20	0.09	0.22	0.07
*Bacillus clausii* KSM-K16	0.46	0.02	0.44	0.04	0.53	−0.05	0.57	−0.09
*Bacillus cohnii* NBRC 15565	0.17	0.09	0.13	0.13	0.15	0.11	0.29	−0.03
*Bacillus drentensis* NBRC 102427	0.28	0.09	0.22	0.15	0.29	0.08	0.31	0.06
*Bacillus firmus* NBRC 15306	0.35	0.08	0.25	0.18	0.31	0.12	0.42	0.01
*Bacillus flexus* Riq5	0.18	0.14	0.13	0.19	0.20	0.12	0.30	0.02
*Bacillus horikoshii* DSM 8719	0.27	0.13	0.16	0.24	0.26	0.14	0.41	−0.01
*Bacillus krulwichiae* NBRC 102362	0.21	0.08	0.18	0.11	0.20	0.09	0.26	0.03
*Bacillus megaterium* WSH-002	0.18	0.14	0.13	0.19	0.18	0.14	0.28	0.04
*Bacillus methanolicus* MGA3	0.33	0.03	0.25	0.11	0.32	0.04	0.41	−0.05
*Bacillus niacini* NBRC 15566	0.23	0.10	0.16	0.17	0.21	0.12	0.42	−0.09
*Bacillus novalis* NBRC 102450	0.33	0.08	0.32	0.09	0.32	0.09	0.54	−0.13
*Bacillus pseudofirmus* OF4	0.21	0.13	0.14	0.20	0.23	0.11	0.37	−0.03
*Bacillus pseudomycoides* DSM 12442	0.18	0.07	0.12	0.13	0.16	0.09	0.31	−0.06
*Bacillus pumilus* NJ-V2	0.24	0.16	0.19	0.21	0.33	0.07	0.40	0.00
*Bacillus simplex* SH-B26	0.26	0.14	0.15	0.25	0.29	0.11	0.44	−0.04
*Bacillus soli* NBRC 102451	0.33	0.07	0.29	0.11	0.29	0.11	0.35	0.05
*Bacillus vallismortis* DV1-F-3	0.00	0.49	0.31	0.18	0.39	0.10	0.45	0.04
*Bacillus vireti* LMG 21834	0.32	0.09	0.32	0.09	0.31	0.10	0.49	−0.08
*Bdellovibrio bacteriovorus* HD100	0.29	0.33	0.24	0.38	0.48	0.14	0.00	0.62
*Beggiatoa alba* B18LD	0.54	−0.10	0.42	0.02	0.47	−0.03	0.48	−0.04
*Beijerinckia indica indica* ATCC 9039	0.81	−0.07	0.80	−0.06	0.76	−0.02	0.76	−0.02
*Brevibacillus agri* BAB-2500	0.58	0.13	0.45	0.26	0.62	0.09	0.76	−0.05
*Burkholderia ambifaria* IOP40-10	0.86	0.04	0.81	0.09	0.86	0.04	0.94	−0.04
*Burkholderia anthina* AZ-4-2-10-S1-D7	0.87	0.05	0.85	0.07	0.86	0.06	0.96	−0.04
*Chlorobium phaeovibrioides* DSM 265	0.55	0.05	0.51	0.09	0.55	0.05	0.65	−0.05
*Chromobacterium subtsugae* MWU2387	0.88	0.02	0.80	0.10	0.88	0.02	0.94	−0.04
*Chromobacterium vaccinii* 21-1	0.90	0.00	0.82	0.08	0.84	0.06	0.95	−0.05
*Clostridium acetobutylicum* EA 2018	0.10	0.05	0.05	0.10	0.08	0.07	0.19	−0.04
*Clostridium argentinense* CDC 2741	0.09	0.04	0.12	0.01	0.08	0.05	0.00	0.13
*Clostridium butyricum* JKY6D1	0.08	0.02	0.05	0.05	0.02	0.08	0.05	0.05
*Clostridium cadaveris* NLAE-zl-G419	0.07	0.06	0.05	0.08	0.06	0.07	0.00	0.13
*Clostridium cochlearium* NLAE-zl-C224	0.09	0.03	0.07	0.05	0.07	0.05	0.00	0.12
*Clostridium pasteurianum* DSM 525 = ATCC 6013	0.09	0.08	0.42	−0.25	0.08	0.09	0.09	0.08
*Clostridium scatologenes* ATCC 25775	0.07	0.05	0.43	−0.31	0.04	0.08	0.11	0.01
*Clostridium sporogenes* NCIMB 10696	0.08	0.05	0.43	−0.30	0.06	0.07	0.00	0.13
*Clostridium tetani* 12124569	0.10	0.03	0.00	0.13	0.05	0.08	0.00	0.13
*Desulfobacterium autotrophicum* HRM2, DSM 3382	0.54	0.05	0.71	−0.12	0.55	0.04	0.70	−0.11
*Desulfobacter postgatei* 2ac9	0.55	0.04	0.43	0.16	0.60	−0.01	0.74	−0.15
*Desulfocapsa sulfexigens* DSM 10523	0.33	0.13	0.61	−0.15	0.38	0.08	0.50	−0.04
*Desulfobacula toluolica* Tol2	0.43	0.04	0.23	0.24	0.42	0.05	0.59	−0.12
*Flavobacterium pectinovorum* DSM 6368	0.10	0.16	0.05	0.21	0.14	0.12	0.32	−0.06
*Flavobacterium suncheonense* GH29-5, DSM 17707	0.33	0.16	0.29	0.20	0.47	0.02	0.00	0.49
*Hyphomicrobium denitrificans* 1NES1	0.86	−0.12	0.81	−0.07	0.76	−0.02	0.77	−0.03
*Micromonospora aurantiaca* ATCC 27029	0.91	0.02	0.95	−0.02	0.83	0.10	0.98	−0.05
*Micromonospora carbonacea* DSM 43168	0.93	0.02	0.95	0.00	0.88	0.07	0.98	−0.03
*Micromonospora chokoriensis* DSM 45160	0.89	0.01	0.88	0.02	0.80	0.10	0.94	−0.04
*Micromonospora echinospora* DSM 43816	0.93	−0.01	0.95	−0.03	0.84	0.08	0.96	−0.04
*Micrococcus luteus* NCTC 2665	0.96	−0.01	0.96	−0.01	0.92	0.03	0.98	−0.03
*Micromonospora purpureochromogenes* DSM 43821	0.92	0.02	0.93	0.01	0.83	0.11	0.96	−0.02
*Nitrosomonas communis* Nm2	0.35	0.12	0.28	0.19	0.30	0.17	0.38	0.09
*Nitrosomonas europaea* ATCC 19718	0.39	0.19	0.39	0.19	0.56	0.02	0.56	0.02
*Nitrobacter hamburgensis* X14	0.94	−0.13	0.89	−0.08	0.91	−0.10	0.91	−0.10
*Nitrobacter winogradskyi* Nb-255	0.89	−0.07	0.90	−0.08	0.91	−0.09	0.91	−0.09
*Nocardia cerradoensis* NBRC 101014	0.85	0.03	0.85	0.03	0.91	−0.03	0.90	−0.02
*Nocardia otitidiscaviarum* IFM 11049	0.00	0.89	0.88	0.01	0.84	0.05	0.93	−0.04
*Pseudomonas azotoformans* S4	0.61	0.20	0.61	0.20	0.60	0.21	0.86	−0.05
*Pseudomonas citronellolis* P3B5	0.86	0.07	0.83	0.10	0.85	0.08	0.97	−0.04
*Pseudomonas fluorescens* A506	0.63	0.17	0.60	0.20	0.63	0.17	0.90	−0.10
*Pseudomonas mendocina* NK-01	0.78	0.03	0.68	0.13	0.77	0.04	0.92	−0.11
*Pseudomonas oryzihabitans* USDA-ARS-USMARC-56511	0.81	0.06	0.86	0.01	0.79	0.08	0.89	−0.02
*Pseudomonas putida* 1A00316	0.75	0.12	0.64	0.23	0.74	0.13	0.96	−0.09
*Rhizobium gallicum* IE4872	0.82	−0.06	0.72	0.04	0.71	0.05	0.82	−0.06
*Streptomyces avermitilis* MA-4680	0.88	0.03	0.88	0.03	0.78	0.13	0.95	−0.04
*Streptomyces clavuligerus* ATCC 27064	0.90	0.02	0.86	0.06	0.84	0.08	0.96	−0.04
*Streptomyces hygroscopicus limoneus* KCTC 1717	0.88	0.04	0.90	0.02	0.84	0.08	0.95	−0.03
*Streptomyces noursei* ATCC 11455	0.89	0.03	0.86	0.06	0.84	0.08	0.97	−0.05
*Streptomyces rubidus* CGMCC 4.2026	0.94	0.00	0.93	0.01	0.86	0.08	0.99	−0.05
*Streptomyces scabrisporus* DSM 41855	0.90	0.00	0.90	0.00	0.80	0.10	0.98	−0.08
*Streptomyces vitaminophilus* ATCC 31673	0.94	−0.02	0.94	−0.02	0.86	0.06	0.96	−0.04
*Thiobacillus denitrificans* ATCC 25259	0.93	−0.04	0.92	−0.03	0.91	−0.02	0.93	−0.04
*Vibrio gazogenes* DSM 21264	0.31	0.19	0.26	0.24	0.34	0.16	0.57	−0.07
*Vibrio natriegens* NBRC 15636	0.22	0.20	0.51	−0.09	0.19	0.23	0.44	−0.02

*GC3_avg_-rpoB GC3, GC3_avg_-atpD GC3, GC3_avg_-infB GC3, and GC3_avg_-trpB GC3 denotes the difference in GC3 values between the genomic GC3 content and the corresponding housekeeping gene GC3 content*.

### Comparative CUB Analysis of *atpD* Gene From Soil Bacteria

The codon usage parameters for the *atpD* gene was also found to be similar to that of the *rpoB* gene in terms of Nc value; the lowest Nc value (24.83) of *atpD* was demonstrated by the same organism *Micrococcus luteus* NCTC 2665. *M. luteus* NCTC 2665 thus display a consistency in terms of Nc pattern. The highest Nc value (55.37) of *atpD* was exhibited by *Desulfocapsa sulfexigens* DSM 10523. In this regard *Nitrosomonas* also exhibited a significantly higher Nc value (50.24) for *atpD* gene. While *Flavobacterium pectinovorum* DSM 6368 and *Acidiphilium cryptum* JF-5 were found to reflect the lowest (4%) and the highest (97%) GC3 value, respectively, *Micrococcus luteus* NCTC 2665 demonstrated a significantly high GC3 content (96%). The CUB profile of *atpD* of all the studied taxa is tabulated in Tables 2, 3. The lowest hydrophobicity value for the *atpD* gene product (−0.256) was observed in *Clostridium cochlearium* NLAE-zl-C224 and the highest value (0.015) was found to be demonstrated by *Beijerinckia indica indica* ATCC 9039, a Gram negative free living soil dweller. A scattered plot showing the distribution of hydrophobicity values of the protein encoded by *atpD* gene is given in Figure 1.

### Comparative CUB Analysis of *infB* Gene From Soil Bacteria

In accordance with the findings of *rpoB* and *atpD, Micrococcus luteus* NCTC 2665 was found to display the lowest Nc and highest GC3 content for *infB*. Similar to *atpD*, the lowest GC3 content was demonstrated by *Clostridium*. But highest Nc for *infB* was demonstrated by *Desulfobacterium autotrophicum HRM2, DSM 3382*. The lowest and most negative hydrophobicity value of *infB* gene product (−0.729) was observed in *Brevibacillus agri* BAB-2500. The CUB profile of *infB* of all the studied bacteria is given in Tables 2, 3. In terms of hydrophobicity, *Bacillus megaterium* WSH-002 was found to display the highest hydrophobicity value (−0.168). A scattered plot showing the distribution of hydrophobicity values of *infB* gene products is given in Figure 1.

### Comparative CUB Analysis of *trpB* Gene From Soil Bacteria

The CUB profile demonstrated by *trpB* was found to be relatively different from the rest of the housekeeping genes. The lowest Nc value (Nc = 25.44) was exhibited by *Pseudomonas putida 1A00316* whereas the highest Nc value (Nc = 57.20) was depicted in *Bacillus horikoshii* DSM 8719. In terms of GC3 content, the lowest and highest GC3 was depicted by *Clostridium butyricum* JKY6D1 (GC3 = 5%) and *Acidiphilium cryptum* JF5 (GC3 = 99%). The CUB profile of *trpB* of the 92 bacterial species is given in Tables 2, 3. The lowest hydrophobicity of (−0.348) was detected in *Clostridium butyricum* JKY6D1 and the highest in different species of the genus *Micromonospora, Nocardia cerradoensis* NBRC 101014 *and Streptomyces scabrisporus* DSM 41855. A scattered plot showing the distribution of hydrophobicity values of the protein encoded by *trpB* gene is given in Figure 1.

Out of the four housekeeping genes, *trpB* was found to demonstrate relatively greater fluctuation in terms of codon bias. The genic Nc of *trpB* were found to be higher than the mean genomic Nc, in 20 out of the 92 soil bacteria considered in this study. Moreover, the reverse case scenario of lower genic Nc of *trpB* in comparison to the genomic Nc was found in about 70% of the organisms. Several species of *Bacillus, Nitrosomonas, Acidithiobacillus* and others displayed this trend. In addition, the genic Nc of *infB* was found to be greater than the mean genomic Nc in almost 8% of the studied organisms. The *rpoB* and *atpD* gene in a small number of organisms was found to display Nc value higher than the mean genomic Nc. On the contrary, for each and every housekeeping gene from the different soil bacteria considered in this study, the genic Nc was found to be much lower than the mean genomic Nc. The range of such deviation was found to be almost 30%. One such example is *Vibrio natriegens* NBRC 15636 which showed 30% deviation in *rpoB*, 27% in *infB*, and negligible deviation in *trpB*. For each of the four housekeeping genes, we have also calculated the percentage of genes in the genome of each organism which is below the genic Nc value of the housekeeping genes (data shown in Supplementary Table 4). Our results show that for the *rpoB* and *infB* gene, *Beggiatoa alba* B18LD had the highest percentage of genes (75.07% for *rpoB* and 76.63% for *infB*) below the genic Nc. In case of the *atpD* gene, *Clostridium argentinense* CDC 2741 had 83.64% of its genes below the genic Nc whereas for the *trpB* gene, *Acidithiobacillus ferrivorans* SS3 demonstrated a remarkably higher number of genes (86.88%) having Nc less than that of *trpB*. The results of *trpB* gene further shows that a large number of bacteria have a greater percentage of genes with Nc values less than the Nc of *trpB*. This finding indicates the aberrant nature of codon bias in *trpB* which being a key housekeeping gene demonstrates a significantly reduced codon usage bias. We observed that in most of the soil bacteria having fewer number of genes below the genic Nc of the *rpoB* gene, also had lesser percentage of genes below the genic Nc of the housekeeping genes *infB* and *atpD*. In the case of *trpB* gene, barring a few organisms, such as *Bdellovibrio bacteriovorus* HD100, *Clostridium cadaveris* NLAE-zl-G419, *Streptomyces vitaminophilus* ATCC 31673, *Rhizobium gallicum* IE4872, *Acidiphilium multivorum* AIU301, and *Nitrobacter winogradskyi* Nb-255 most of the bacteria did not have a large percentage of genes in their genome which is below the genic Nc of the remaining housekeeping genes.

In terms of GC3 content, *trpB* exhibited a relatively greater fluctuation. In almost 70% of the soil bacterial species higher genic GC3 content was observed in comparison to the genomic GC3 value. The gene *atpD* showed similar type of deviation in 25 species. These include *Clostridium scatologenes* ATCC 25775, *C. sporogenes* NCIMB 10696, *Desulfocapsa sulfexigens* DSM 10523*, Desulfobacterium autotrophicum* HRM2, DSM 3382*, Vibrio natriegens* NBRC 15636*, Acidithiobacillus ferrivorans* SS3*, Nitrobacter winogradskyi* Nb-255*, N. hamburgensis* X14*, Acidiphilium cryptum* JF-5, and *Hyphomicrobium denitrificans* 1NES1. The common features shared by these organisms are that these are Gram negative (except *Clostridium*), free living soil bacteria. Out of these *Acidithiobacillus ferrivorans* SS and *Acidiphilium cryptum* JF-5 are from acidic environment. Both the *infB* and *rpoB* gene also reflected similar trend. On the contrary, in some species in which genomic GC3 content was found to be much higher than the GC3 content of the housekeeping genes, and in all the concerned housekeeping genes such a deviation was found to exist on a massive scale. For example, the *atpD* codon profile of *Flavobacterium pectinovorum* DSM 6368 was found to display a whopping 81% deviation.

### Relative Hydrophobicity Analysis of Housekeeping Gene Products From Soil Bacteria

A comparison of the hydrophobicity of the different housekeeping gene products revealed an interesting pattern in the 92 soil bacterial species (Figure 1). We observed that the hydrophobicity of the protein coded by *rpoB* gene demonstrates a tight clustering compared to the products of the other three housekeeping genes suggesting that in the soil bacteria the hydrophobicity of the housekeeping gene product *rpoB* is relatively conserved in comparison to *atpD, infB* and *trpB*. This is understandable since *rpoB* is responsible for coding the β subunit of bacterial RNA polymerase which is a key component of the genetic information processing pathway, and regions of the protein susceptible to mutations are characteristically safeguarded (Vos et al., 2012) for preserving function of the protein. In contrast to *rpoB, atpD*, and *trpB*, the *infB* gene products was found to demonstrate a relatively fluctuating degree of hydrophobicity with values ranging from −0.168 to −0.729 within the soil bacteria. In spite of the diversity of the soil bacteria and the dissimilar functional nature of the housekeeping gene products a greater fraction of the coding sequences in the 92 species were found to display a hydrophilic nature as depicted in Figure 1. This clearly demonstrates that the 92 soil bacterial species are unified by a relatively hydrophilic character of their housekeeping gene products although they grow and survive in soil types having different physico-chemical properties.

A comprehensive Spearman's rank-order correlation study between Nc, GC3, length of the housekeeping gene, and hydrophobicity of each of the housekeeping gene product was thoroughly carried out. Our objective was to understand the underlying codon usage trend and ORF structuring of each of the housekeeping gene considered in this study, and comprehend whether each gene carries an underlying signature in terms of nucleotide structuring of the coding sequences. For all the four housekeeping genes, Nc was found to be significantly anti-correlated with GC3 (for *rpoB* ρ = −0.649; for *atpD* ρ = −0.633; *infB* ρ = −0.54; for *trpB* ρ = −0.766; at *p* < 0.01 level). The negative correlation between Nc and length was demonstrated by *trpB* (ρ = −0.457, *p* < 0.01)*, infB* (ρ = −0.257, *p* < 0.01), and *atpD* (ρ = −0.197, *p* < 0.01). These suggest that in the later three housekeeping genes, the codon bias is positively correlated with increasing gene length. Similarly, the Nc and hydrophobicity was observed to be significantly anti-correlated for the gene *rpoB* (ρ = −0.303, *p* < 0.01) and *trpB* (ρ = −0.418, *p* < 0.01) in the soil bacteria. Except *rpoB*, the rest of the three housekeeping genes were found to show significant positive correlation between GC3 and length suggesting the fact that G and C ending codons are preferentially more favored with increasing ORF length. GC3 was also found to display a significant positive correlation with hydrophobicity for the gene *rpoB* (ρ = 0.509, *p* < 0.01) and *trpB* (ρ = 0.467, *p* < 0.01). This is expected as the presence of G or C residue at the 3′ position of a codon is a feature of hydrophobic amino acids. The correlation between length and hydrophobicity was also found to be significantly positive in case of *rpoB* (ρ = 0.32, *p* < 0.01) and *trpB* (ρ = 0.308, *p* < 0.01), whereas *infB* (ρ = −0.337, *p* < 0.01) was found to depict a negative association. Among the four housekeeping genes, *trpB* was found to depict a significant correlation between all the four parameters studied. This appears to be a completely atypical profile when compared to the other three housekeeping genes considered in this analysis. This aberrant profile of *trpB* could be further validated by studying its phylogenetic affinities which has been discussed in the succeeding sections of this study.

Kruskal-Wallis one way analysis of variance on ranks (Daniel, 1990) was performed to scrutinize whether the different codon usage parameters in the housekeeping genes selected in this study have a signature trend utilizing Nc, GC3, gene length and hydrophobicity. GC3, gene length, and hydrophobicity were all found to display a unique trend making it possible to delineate the four genes in terms of their signature GC3 content (*H* = 7.903, *df* = 3, *p* < 0.01), gene length (*H* = 321.508, *df* = 3, *p* < 0.01), and hydrophobicity (*H* = 268.193, *df* = 3, *p* < 0.01). However, the housekeeping genes did not demonstrate any signature trend in terms of their Nc values (*H* = 7.166, *df* = 3, *p* = 0.067).

Mann-Whitney Rank sum test utilizing Nc, GC3, gene length and hydrophobicity was carried out to find out whether the Gram nature of the bacterial species have a significant effect on the codon usage profile leading to a genic signature pattern. The test results suggested that in terms of GC3 (*U* = 700; *p* < 0.01), gene length (*U* = 82; *p* < 0.01) and hydrophobicity (*U* = 551; *p* < 0.01), it is possible to substantially delineate the *rpoB* gene between Gram negative and Gram positive bacteria. The results further indicate that the GC3 (*U* = 663; *p* < 0.01) and gene length (*U* = 469.50; *p* < 0.01) of the housekeeping gene *infB* is also clearly distinguishable between the Gram negative and Gram positive bacterial species. The GC3 (*U* = 770; *p* < 0.05) and hydrophobicity (*U* = 353; *p* < 0.01) of the protein encoded by the housekeeping gene *atpD* also appeared to be distinct and depict a genic signature among the two bacterial groups. The Nc of all the four housekeeping genes did not show any characteristic delineation based on the results of the Mann Whitney rank sum test between the Gram negative and Gram positive bacterial species. Apart from *trpB*, the GC3 profile of all the remaining housekeeping genes were found to be distinct among the Gram positive and Gram negative bacteria. The Mann-Whitney rank sum test results demonstrated that out of the four housekeeping genes, the Nc (*U* = 893; *p* >> 0.05), GC3 (*U* = 844.50; *p* >> 0.05), gene length (*U* = 883.50; *p* >> 0.05) of *trpB* and hydrophobicity (*U* = 881; *p* >> 0.05) of *trpB* gene product did not depict any statistically significant difference between Gram positive and Gram negative bacterial species. Our results thus further corroborates the finding that the housekeeping gene *trpB* demonstrates an aberrant codon usage profile in comparison to the rest of the housekeeping genes considered in this study. The *trpB* gene encodes the β subunit of the enzyme tryptophan synthase, which is a pyridoxal 5′-phosphate-dependent α*ββα* multi enzyme complex, responsible for catalyzing the final two steps of tryptophan biosynthesis (Yanofsky, 2001; Ishida et al., 2002; Dunn et al., 2008; Raboni et al., 2009). This enzyme is bestowed with certain unique features, such as dual catalytic ability, substrate channeling (Leopoldseder et al., 2006; Raboni et al., 2009) and the *trpB* gene might have been subjected to gene duplication, fusion, loss as well as parallel evolution in some archaea and eubacteria (Xie et al., 2001; Leopoldseder et al., 2006). Therefore, it can be construed that the *trpB* gene with its inconsistent codon usage pattern may be deemed as an odd gene in terms of its codon usage pattern residing within the genome of many soil dwelling bacteria.

### Analysis of Nc Plot

**Figure 2:**
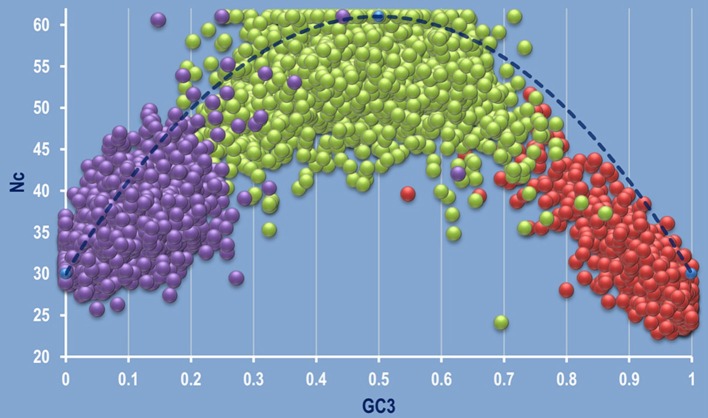
A combined genomic Nc plot utilizing all the coding sequences of the whole genomes depicting the three typical mode of aggregation of coding sequences. Left centric aggregation represented by *Clostridium butyricum* JKY6D1 (in purple), mid centric aggregation shown in green by *Nitrosomonas communis* Nm2, and right centric aggregation depicted by *Micrococcus luteus* NCTC 2665, shown in red. The dashed blue line represents the null hypothesis curve which suggests that codon usage bias is solely due to mutation and not selection (Wright, 1990).

The correlation between Nc and GC3 was plotted on a graph called Nc plot (Wright, 1990) for the 92 species considered in this study. Our previous study has shown that the Nc plots can be used as a valuable tool to detect intra- and inter-specific/genic synonymous codon usage patterns (Pal et al., 2015). Analyzing the Nc plots, three types of gene clustering on the Nc plot was evident—left centric, mid centric and right centric aggregation of coding sequences as shown in Figure 2. Several genera like *Acidiphilium, Alcaligenes, Agrobacterium, Actinoalloteichus, Acidobacterium, Achromobacter, Acidovorax, Azotobacter, Burkholderia, Hyphomicrobium, Micrococcus, Micromonospora, Nocardia, Streptomyces, Rhizobium, Thiobacillus*, and *Nitrobacter* exhibited right centric aggregation of the coding sequences on the Nc plot. But some members of the genera *Acidithiobacillus, Acidocella, Chromobacterium*, and some species, such as *Achromobacter xylosoxidans* A8, *Beijerinckia indica indica* ATCC 9039 showed an aberrant mid centric aggregation with right shift (Supplementary Figure 1). It was observed that the left centric Nc plot is the general feature of the genera *Clostridium* and *Bacillus*. The organism *Nitrosomonas communis* Nm2*, Nitrosomonas europaea* ATCC 19718, *Desulfobacter postgatei* 2ac9*, Chlorobium phaeovibrioides* DSM 265*, Desulfobacterium autotrophicum* HRM2 DSM 3382*, Desulfobacula toluolica* Tol2*, Vibrio gazogenes* DSM 21264 demonstrated a mid-centric aggregation of the coding sequences. All these species are free living, Gram negative soil dwellers. An aberrant pattern was found to be displayed by the genus *Flavobacterium* where two species were found to display two distinct type of clustering on the Nc plot. *F. suncheonense* GH29-5, DSM 17707 demonstrated a mid to left centric aggregation of the coding sequences whereas *F. pectinovorum* DSM 6368 exhibited a left centric aggregation (Supplementary Figure 2). The members of the genus *Pseudomonas* like *P. azotoformans* S4, *P*. *fluorescens* A506, *P. putida* demonstrated some variation in clustering pattern on the Nc plot with majority of the species characterized by a right centric aggregation on the Nc plot. The coding sequences of *Acinetobacter calcoaceticus* PHEA-2 was found to demonstrate a typical left centric aggregation on the Nc plot.

**Figure 3:**
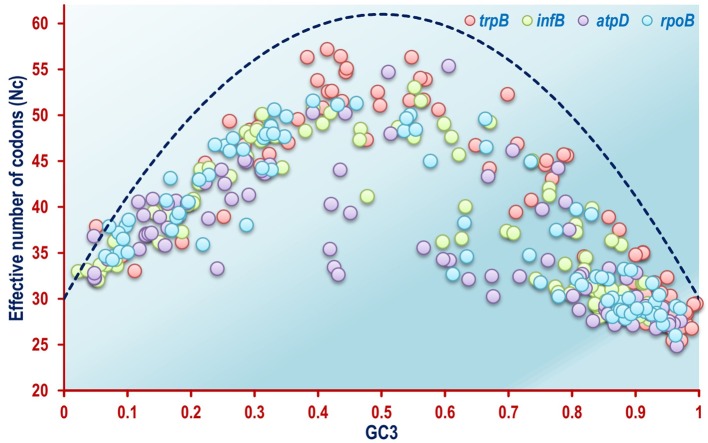
A combined Nc plot of the four housekeeping genes *rpoB, atpD, infB*, and *trpB* from the 92 soil bacterial species included in this study, depicting selectional pressure as a major unifying force in shaping codon usage pattern. The dashed blue line represents the null hypothesis curve which suggests that codon usage bias is solely due to mutation and not selection (Wright, 1990).

The Nc plots of the four housekeeping genes *atpD, infB, rpoB*, and *trpB* revealed a different scenario as a whole. In case of the *rpoB* gene, which codes for the β-subunit of DNA dependent RNA polymerase enzyme in bacteria, most of the organisms were found to form scattered clusters all throughout the Nc plot (Figure 3). The Nc plots based on *atpD, infB* and *trpB* coding sequences exhibited a comparable aggregation pattern as shown in Figure 3. The mechanistic forces shaping codon usage can also be detected utilizing an Nc plot (Wright, 1990; Khandia et al., 2019). Barring a mere 1.4% out of the total 360 coding sequences of the four housekeeping genes, all the remaining coding sequences of the *rpoB*, atpD*, infB*, and *trpB* gene were found to fall below the null hypothesis curve of the Nc plot in Figure 3 indicating selection pressure as a key element shaping codon usage pattern in the housekeeping genes of a majority of the soil bacterial species considered in this study.

### Molecular Phylogenetic Analysis

To further comprehend the potential relationships between the different soil dwelling bacteria, a comprehensive molecular phylogenetic analysis was carried out using sequences of complete 16S rRNA gene and the four housekeeping genes.

### Phylogenetic Analysis Based on 16S rRNA Gene Sequences

**Figure 4:**
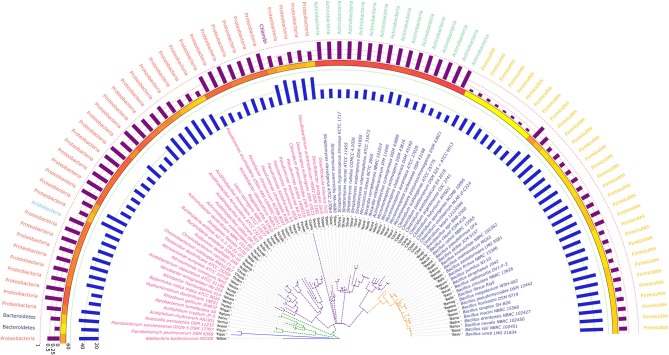
A phylogenetic tree showing the relationship between the soil bacterial species considered in this study based on 16S rRNA gene sequences along with Gram nature, taxonomic position and codon usage annotation data. The name of the species have been depicted in color corresponding to its Gram nature with magenta and blue representing Gram negative and positive nature, respectively. The outermost semicircle with magenta bars represents the genomic GC3 while the innermost semicircle with blue bars represents the genomic Nc. The middle strip with yellow to red color gradient depicts the genomic GC content with red representing maximum GC content. The evolutionary history was inferred by using the Maximum Likelihood method based on the Kimura 2-parameter model (Kimura, 1980). The bootstrap consensus tree inferred from 1,000 replicates is taken to represent the evolutionary history of the taxa analyzed (Felsenstein, 1985). The tree with the highest log likelihood (−6,331.0306) is shown. Initial tree(s) for the heuristic search were obtained automatically by applying Neighbor-Join and BioNJ algorithms to a matrix of pairwise distances estimated using the Maximum Composite Likelihood (MCL) approach, and then selecting the topology with superior log likelihood value. A discrete Gamma distribution was used to model evolutionary rate differences among sites [five categories (+G, parameter = 0.5623)]. The rate variation model allowed for some sites to be evolutionarily invariable ([+I], 40.6240% sites). The tree is drawn to scale, with branch lengths measured in the number of substitutions per site. All positions containing gaps and missing data were eliminated. There were a total of 357 positions in the final dataset. Evolutionary analyses were conducted in MEGA6 (Kumar et al., 2008). The visualization and annotation of the phylogenetic tree was done using iTOL ver. 4.4.2 (Letunic and Bork, 2007).

The phylogram based on 16S rRNA gene sequences (Figure 4), depicted that species belonging to the same genera share more than 95% sequence similarity and cluster nearly together. This is in line with the traditional taxonomic standing. Bootstrap values indicate that most branches in the dendrogram are highly significant, although some exception exists (data provided as Supplementary File in Newick format). A prominent and close association of the organisms belonging to the same taxonomic classes, were also visible, but with certain exceptions. The genus *Bdellovibrio* belonging to the class Proteobacteria showed proximity with the genus *Flavobacterium* under the class Bacteroidetes. On the contrary, at the sub-class level too, some variations were detected. Some genera belonging to the subclass Gammaproteobacteria like *Vibrio, Beggiatoa, Acinetobacter, Pseudomonas*, and *Azotobacter* formed cluster with species belonging to the sub-class Betaproteobacteria. Above all, the class Firmicutes, Actinobacteria, Chlorobi, Proteobacteria showed similar ancestral origin but not through the dichotomous branching rather through a polytomy as seen in the Figure 4. The annotated phylogenetic tree inferred utilizing the 16S rRNA gene sequences from the 92 soil bacteria is shown in Figure 4. The raw tree file with bootstrap support in Newick format is provided as Supplementary File.

### Phylogenetic Analysis of Soil Bacteria Based on Housekeeping Genes

Housekeeping genes constitute an integral component of an organism's genome. Functioning in a coordinated fashion the housekeeping genes are involved with either metabolic functions, genetic information processing or environmental signal processing and are generally organized into operational units or operons in prokaryotes like bacteria. The antiquity of these housekeeping genes predates their function and hence these can be used as excellent markers to deduce the relationship between organisms on the functional basis. The use of 16S rRNA as a molecular chronometer, and inference of phylogeny based on it have been a major milestone in molecular taxonomy (Ludwig and Schleifer, 1994). The use of 16S rRNA based phylogeny as a backdrop against the phylogeny constructed from housekeeping genes thus, should be extremely vital in shedding new light on the functional relationship amongst soil dwelling bacteria.

### Phylogenetic Analysis Based on *rpoB* Housekeeping Gene

**Figure 5:**
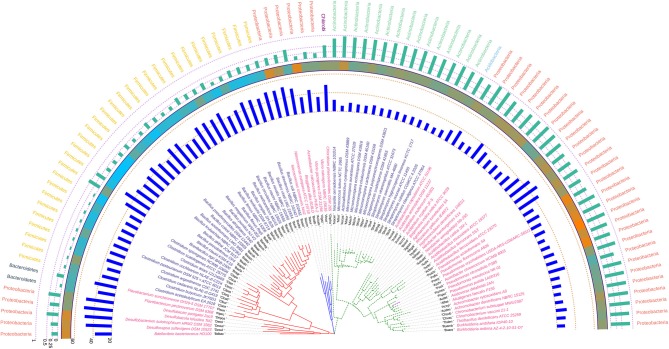
Phylogenetic tree showing the relationship between the soil bacterial species considered in this study based on *rpoB* gene sequences along with Gram nature, taxonomic position and codon usage annotation data. The name of the species have been depicted in color corresponding to the Gram nature with magenta and blue representing Gram negative and positive, respectively. The outermost semicircle with green bars represents the GC3 content of *rpoB* sequences while the innermost semicircle with blue bars represents the Nc of the *rpoB* coding sequences. The middle strip with cyan to orange color gradient depicts the variation in hydrophobicity of the protein encoded by *rpoB* coding sequences. The evolutionary history was inferred by using the Maximum Likelihood method based on the General Time Reversible model (Nei and Kumar, 2000). The bootstrap consensus tree inferred from 1,000 replicates is taken to represent the evolutionary history of the taxa analyzed (Felsenstein, 1985). The tree with the highest log likelihood (−73,689.4674) is shown. Initial tree(s) for the heuristic search were obtained automatically by applying Neighbor-Join and BioNJ algorithms to a matrix of pairwise distances estimated using the Maximum Composite Likelihood (MCL) approach, and then selecting the topology with superior log likelihood value. A discrete Gamma distribution was used to model evolutionary rate differences among sites [five categories (+G, parameter = 0.7988)]. The rate variation model allowed for some sites to be evolutionarily invariable ([+I], 22.2913% sites). The tree is drawn to scale, with branch lengths measured in the number of substitutions per site. All positions containing gaps and missing data were eliminated. There were a total of 1,726 positions in the final dataset. Evolutionary analyses were conducted in MEGA6 (Kumar et al., 2008). The visualization and annotation of the phylogenetic tree was done using iTOL ver. 4.4.2 (Letunic and Bork, 2007).

The phylogenetic tree inferred using *rpoB* demonstrated a polytomy from inception but the aggregation of the species was found to be quite in congruence with that of the 16S rRNA gene based tree suggesting the relative absence of horizontal gene transfer and homologous recombination in course of evolution in these bacteria. The gene *rpoB* codes for a fundamental component of bacterial RNA polymerase, the β-subunit. This is a key component of the genetic information processing pathway and may be also considered as one of the most antique nucleic acid polymerizing enzyme. Hence, the congruence of the 16S rRNA and *rpoB* gene is quite justified looking at the antiquity of both these genes. When compared with the 16S rRNA gene based phylogenetic tree, we found that the group containing Betaproteobacteria in the 16S rRNA based tree ruptures into separate clusters based on their *rpoB* sequence suggesting diverse phylogenetic affinities. *Azotobacter chroococcum* was found to share clade with species of *Pseudomonas* having similar Nc, GC3 and hydrophobicity profile. The degree of codon usage bias variation in *rpoB* as evident from the Nc values of the genus *Bacillus* was found to be significant in comparison to the genera *Clostridium, Pseudomonas, Streptomyces*, and *Micromonospora*, all of which have a codon biased *rpoB*. The annotated phylogenetic tree inferred utilizing the *rpoB* coding sequences along with codon usage and taxonomic data from the studied soil bacteria is shown in Figure 5.

### Phylogenetic Analysis Based on *atpD* Housekeeping Gene

**Figure 6:**
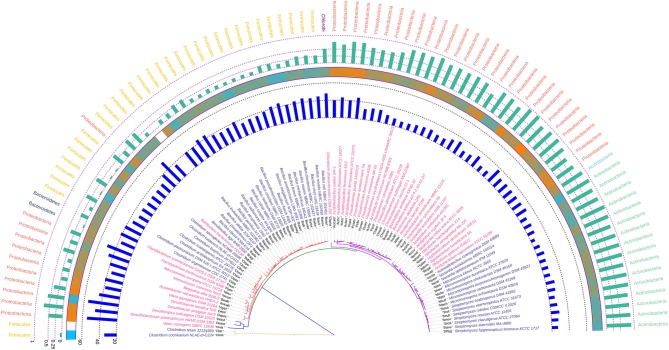
Phylogenetic tree showing the relationship between the soil bacterial species considered in this study based on *atpD* gene sequences along with Gram nature, taxonomic position and codon usage annotation data. The name of the species have been depicted in color corresponding to its Gram nature with magenta and blue representing Gram negative and positive, respectively. The outermost semicircle with green bars represents the GC3 content of *atpD* sequences while the innermost semicircle with blue bars represents the Nc of the *atpD* coding sequences. The middle strip with cyan to orange color gradient depicts the variation in hydrophobicity of the protein encoded by the *atpD* coding sequences. The evolutionary history was inferred by using the Maximum Likelihood method based on the General Time Reversible model (Nei and Kumar, 2000). The bootstrap consensus tree inferred from 1,000 replicates is taken to represent the evolutionary history of the taxa analyzed (Felsenstein, 1985). Initial tree(s) for the heuristic search were obtained automatically by applying Neighbor-Join and BioNJ algorithms to a matrix of pairwise distances estimated using the Maximum Composite Likelihood (MCL) approach, and then selecting the topology with superior log likelihood value. A discrete Gamma distribution was used to model evolutionary rate differences among sites [five categories (+G, parameter = 0.9877)]. The rate variation model allowed for some sites to be evolutionarily invariable ([+I], 6.2680% sites). All positions containing gaps and missing data were eliminated. There were a total of 1,123 positions in the final dataset. Evolutionary analyses were conducted in MEGA6 (Kumar et al., 2008). The visualization and annotation of the phylogenetic tree was done using iTOL ver. 4.4.2 (Letunic and Bork, 2007).

The phylogeny inferred utilizing *atpD* was found to be quite unique with respect to the positioning of the two Clostridia species, *C. cochlearium* NLAE-zl-C224 and *C. tetani* 12124569 in relation to the other seven species of the genera. Though the phylogeny of the *atpD* gene in the soil bacteria suggests least amount of evolutionary divergence as suggested by the branch lengths in comparison to the other three housekeeping genes but, *C. cochlearium* NLAE-zl-C224 and *C. tetani* 12124569 were found to be the most evolutionary distant ones in comparison to the other bacterial species. Similar to what we have seen in *rpoB, Azotobacter chroococcum* was found to share clade with species of *Pseudomonas* with similar Nc and hydrophobicity profile. The species of *Micromonospora* and *Streptomyces* were found to be consistent both in terms of their codon usage and phylogenetic relatedness. The codon usage profile of members of the genus *Bacillus* was found to be much more uniform in comparison to *rpoB*. The *atpD* coding sequence of *Bdellovibrio bacteriovorous* HD100 was found to be peculiar since it made the sole Gram negative bacterium to share clade with a host of Gram positive bacterial genera, such as *Clostridium* and *Bacillus*. The degree of codon bias of *atpD* sequence of *Bdellovibrio* as depicted by Nc was also found to be similar to some of the *Clostridium* species. In the *atpD* based evolutionary tree, bacteria belonging to Firmicutes closely resembled the members of Proteobacteria and hence appears to intermingle with each other forming a cluster. The annotated phylogenetic tree inferred utilizing the *atpD* coding sequences along with codon usage and taxonomic data from the studied soil bacteria is shown in Figure 6.

### Phylogenetic Analysis Based on *infB* Housekeeping Gene

**Figure 7:**
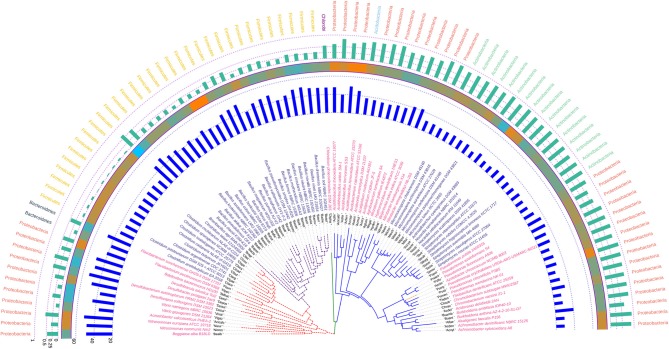
Phylogenetic tree showing the relationship between the soil bacterial species considered in this study based on *infB* gene sequences along with Gram nature, taxonomic position and codon usage annotation data. The name of the species has been depicted in color corresponding to its Gram nature with magenta and blue representing Gram negative and positive, respectively. The outermost semicircle with green bars represents the GC3 content of *infB* sequences while the innermost semicircle with blue bars represents the Nc of the *infB* coding sequences. The middle strip with cyan to orange color gradient depicts the variation in hydrophobicity of the protein encoded by *infB* coding sequences. The evolutionary history was inferred by using the Maximum Likelihood method based on the General Time Reversible model (Nei and Kumar, 2000). The bootstrap consensus tree inferred from 1,000 replicates is taken to represent the evolutionary history of the taxa analyzed (Felsenstein, 1985). The tree with the highest log likelihood (−84,778.7972) is shown. Initial tree(s) for the heuristic search were obtained automatically by applying Neighbor-Join and BioNJ algorithms to a matrix of pairwise distances estimated using the Maximum Composite Likelihood (MCL) approach, and then selecting the topology with superior log likelihood value. A discrete Gamma distribution was used to model evolutionary rate differences among sites [five categories (+G, parameter = 1.1105)]. The rate variation model allowed for some sites to be evolutionarily invariable ([+I], 16.2594% sites). The tree is drawn to scale, with branch lengths measured in the number of substitutions per site. All positions containing gaps and missing data were eliminated. There were a total of 1,617 positions in the final dataset. Evolutionary analyses were conducted in MEGA6 (Kumar et al., 2008). The visualization and annotation of the phylogenetic tree was done using iTOL ver. 4.4.2 (Letunic and Bork, 2007).

The phylogenetic tree inferred utilizing the *infB* coding sequences of the soil bacteria demonstrated absence of polytomy and consistency in the clustering pattern of the different species of the genera particularly *Pseudomonas, Streptomyces, Micromonospora, Clostridium*, and *Bacillus*. All the clades of the *infB* based tree were found to be dichotomous with a relatively short evolutionary distance indicating relatedness and uniformity. The codon usage profile of the *infB* coding sequences in these genera was also found to be consistent except in some species of *Bacillus* like *B. vallismortis* DV1-F-3, *B. pumilus* NJ-V2, *B. atrophaeus* 1942, *B. vireti* LMG 21834, and *B. novalis* NBRC 102450 which depicted a higher Nc value for *atpD* suggesting relatively lower codon bias. Similar to the phylogenetic trees based on the housekeeping genes *rpoB* and *atpD, Azotobacter chroococcum* was found to cluster with species of *Pseudomonas* on the basis of the *infB* coding sequence and show similar codon usage profile comparable to some species of *Pseudomonas*. The annotated phylogenetic tree inferred utilizing the *infB* coding sequences along with codon usage and taxonomic data from the studied soil bacteria is shown in Figure 7.

### Phylogenetic Analysis Based on *trpB* Housekeeping Gene

**Figure 8:**
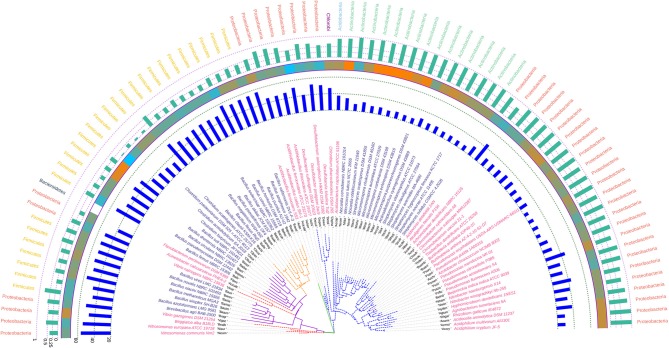
Phylogenetic tree showing the relationship between the soil bacterial species considered in this study based on *trpB* gene sequences along with Gram nature, taxonomic position and codon usage annotation data. The name of the species has been depicted in color corresponding to its Gram nature with magenta and blue representing Gram negative and positive, respectively. The outermost semicircle with green bars represents the GC3 content of *trpB* sequences while the innermost semicircle with blue bars represents the Nc of the *trpB* coding sequences. The middle strip with cyan to orange color gradient depicts the variation in hydrophobicity of the protein encoded by *trpB* coding sequences. The evolutionary history was inferred by using the Maximum Likelihood method based on the General Time Reversible model (Nei and Kumar, 2000). The bootstrap consensus tree inferred from 1,000 replicates is taken to represent the evolutionary history of the taxa analyzed (Felsenstein, 1985). The tree with the highest log likelihood (−57,296.2790) is shown. Initial tree(s) for the heuristic search were obtained automatically by applying Neighbor-Join and BioNJ algorithms to a matrix of pairwise distances estimated using the Maximum Composite Likelihood (MCL) approach, and then selecting the topology with superior log likelihood value. A discrete Gamma distribution was used to model evolutionary rate differences among sites [five categories (+G, parameter = 1.2054)]. The rate variation model allowed for some sites to be evolutionarily invariable ([+I], 14.7706% sites). The tree is drawn to scale, with branch lengths measured in the number of substitutions per site. All positions containing gaps and missing data were eliminated. There were a total of 1,098 positions in the final dataset. Evolutionary analyses were conducted in MEGA6 (Kumar et al., 2008). The visualization and annotation of the phylogenetic tree was done using iTOL ver. 4.4.2 (Letunic and Bork, 2007).

The odd and inconsistent nature of the *trpB* gene as evident from the codon usage study is further corroborated by phylogenetic analysis. A conflicting signal was clearly evident from the phylogeny inferred utilizing *trpB*. Utilizing the 16S rRNA gene tree at the backdrop, significant differences were observed. All the species of the genus *Bacillus* that were grouped together in the 16S rRNA based tree were found to cluster in three separate groups. Some species of *Clostridium, Vibrio* along with *Flavobacterium pectinovorum* DSM 6368 were found to coexist in these clusters. The organism *Bacillus firmus* NBRC 15306 was found to be distantly related from all the other species of *Bacillus* considered in this study. The two species of *Vibrio, V. gazogenes* DSM 21264 and *V. natriegens* NBRC 15636 was also found to segregate into two separate clades. Similarly the species *Streptomyces scabrisporus* DSM 41855 formed a separate clade from the other *Streptomyces* species considered in our study. Besides, the organism *Streptomyces scabrisporus* DSM 41855 was also found to reside with all of the *Micromonospora* species on the *trpB* phylogram indicating monophyletic ancestral origin. The comparative analysis of hydrophobicity of the *trpB* gene gene products demonstrated a similar sort of relationship where *S. scabrisporus* DSM 41855 was found to display hydrophobicity profile in line with *Micromonospora* species. In spite of the conflicting signals and inconsistencies of *trpB*, species belonging to *Clostridium, Pseudomonas*, and *Micromonospora* were found to cluster together on the tree suggesting their phylogenetic closeness. Similar to the other three housekeeping gene based trees, *Azotobacter chroococcum* was found to cluster together with species of *Pseudomonas* sharing similar Nc and hydrophobicity profile. The annotated phylogenetic tree inferred utilizing the *trpB* coding sequences along with codon usage and taxonomic data is shown in Figure 8.

Significant topological differences were observed between the phylogenetic trees based on the housekeeping genes and the standard 16Sr RNA gene based one, suggesting conflicting evolutionary signals. In all of the scenarios, the Proteobacteria was found to be the worst affected phylum. Based on *rpoB*, the members of Proteobacteria were found to be scattered among all the three clades originating from the root whereas the phylogeny inferred utilizing the housekeeping gene *infB* puts the member of the Proteobacteria dispersed between the phyla Firmicutes and Actinobacteria. Consistent with its fluctuations as evident from the codon usage study, the *trpB* based phylogeny dispersed the members of the phylum Proteobacteria to a large extent. The *atpD* based phylogeny also pointed toward the evolutionary distant nature of the Proteobacteria, particularly in the case of *Bdellovibrio* which was found to be the only Gram negative bacterium residing with the Firmicutes like *Bacillus* and *Clostridium*. In almost all the phylograms, species belonging to the same genus revealed a more or less tight clustering with some degree of heterogeneity. The members of the phylum Actinobacteria and Firmicutes were found to be closely associated with each other in all the phylogenetic trees inferred utilizing the housekeeping gene sequences pointing toward the relative absence of conflicting evolutionary signals. In all the phylograms based on both the 16S rRNA and housekeeping genes, the organism *Azotobacter chroococcum* NCIMB 8003 consistently clustered tightly with a variety of *Pseudomonas* species. *P. citronellolis* P3B5 was mostly found to exhibit close proximity with *Azotobacter chroococcum* NCIMB 8003 with significant bootstrap support. Only in case of *trpB* and *infB* this was found to be incongruent. We also observed that for the housekeeping genes *atpD, rpoB*, and *trpB*, the bacteria *Azotobacter chroococcum* NCIMB8003 shared 80–98% identity with various *Pseudomonas* species. In case of the gene *infB*, the nucleotide sequences of *Azotobacter chroococcum* NCIMB was found to share almost 75% identity. The hydrophobicity profile of the protein encoded by the housekeeping gene *rpoB* also demonstrated the proximity between *Azotobacter chroococcum* NCIMB and species of *Pseudomonas*.

## Conclusion

This exhaustive study conducted on 92 bacterial species is one of its kind where a large scale comparative genomic analysis of soil dwelling bacteria utilizing a combination of codon usage analyses and molecular phylogenetics emphasizing on key housekeeping genes was carried out. The soil may be regarded as a treasure trove of microorganisms with a lot remaining to be explored. Our study revealed signature codon usage trend in the 92 soil bacteria where all the housekeeping genes were found to be under selectional pressure. An irregular codon usage profile and conflicting phylogenetic profile was consistently visible in case of the key housekeeping gene *trpB* encoding the beta subunit of the tryptophan synthase enzyme. The presence of conflicting signals with regard to the housekeeping genes in the bacterial phylum Proteobacteria pointed appreciably to the enormous genetic heterogeneity present within the group which was further corroborated by the codon usage analyses study. The taxonomic positioning of the organism *Azotobacter chroococcum* NCIMB with the Pseudomonads was also a major taxonomic deviation but the signal was consistent and further amplified by all the housekeeping genes measured in this study. Bacterial phylogeny is a controversial issue abetted by the presence of lateral gene transfer and recombination events, and among the soil bacteria, the members of Proteobacteria were found to be the most affected in contrast to that of the members of the phylum Actinobacteria and Firmicutes, a fact also supported by codon usage analysis.

## Data Availability Statement

All datasets generated for this study are included in the article/Supplementary Material.

## Author Contributions

AP conceived and designed the subject with substantial contribution from JS and PM in analysis and interpretation of data. AP and JS wrote the first draft of the manuscript and carried out the statistical analysis. JS, BS, MP, and VR retrieved and analyzed the data and generated the tables and figures under supervision of AP and PM. BS, MP, and VR assisted in writing. All the authors approved final version of the manuscript, and agreed to be accountable for its contents.

### Conflict of Interest

The authors declare that the research was conducted in the absence of any commercial or financial relationships that could be construed as a potential conflict of interest.

## Supplementary Material

Supplementary Figure 1 A genomic Nc plot demonstrating aberrant mid centric aggregation of coding sequences with right shift in the bacterial species *Achromobacter xylosoxidans* A8 (shown in red) and *Beijerinckia indica indica* ATCC 9039 (shown in green). The dashed cyan line represents the null hypothesis curve which suggests that codon usage bias is solely due to mutation and not selection (Wright, 1990).

Supplementary Figure 2 A genomic Nc plot demonstrating aberrant inter specific variation in codon usage pattern in the genus *Flavobacterium*. *F. suncheonense* (shown in red) demonstrates a mid to left centric aggregation of the coding sequences whereas *F. pectinovorum* exhibits a left centric aggregation of the coding sequences (shown in green). The dashed black line represents the null hypothesis curve which suggests that codon usage bias is solely due to mutation and not selection (Wright, 1990).

Supplementary Table 1 A detailed list of the 92 soil bacterial species studied along with their NCBI reference sequence number, abbreviated name, Gram nature, habitat and source of isolation.

Supplementary Table 2 A detailed list showing the genome size (DNA total bases), coding bases, G+C bases, percentage of coding bases and GC content of the genome in the 92 soil bacterial species considered in this study.

Supplementary Table 3 Results of the correlation analysis between the different codon usage parameters at the whole genome level in the 92 soil bacterial species considered in this study.

Supplementary Table 4 A list showing the genomic gene count along with the percentage (%age) of genes in the genome of the 92 soil bacterial species considered in this study that is below the genic Nc value of the housekeeping genes *rpoB, atpD, infB*, and *trpB*.
