# Supplementary figures and images for: Comparative Genomic Analysis of Soil Dwelling Bacteria Utilizing a Combinational Codon Usage and Molecular Phylogenetic Approach Accentuating on Key Housekeeping Genes
Source: Front Microbiol. 2019 Dec 17;10:2896. doi: 10.3389/fmicb.2019.02896 (PMC6928123; doi:10.3389/fmicb.2019.02896)

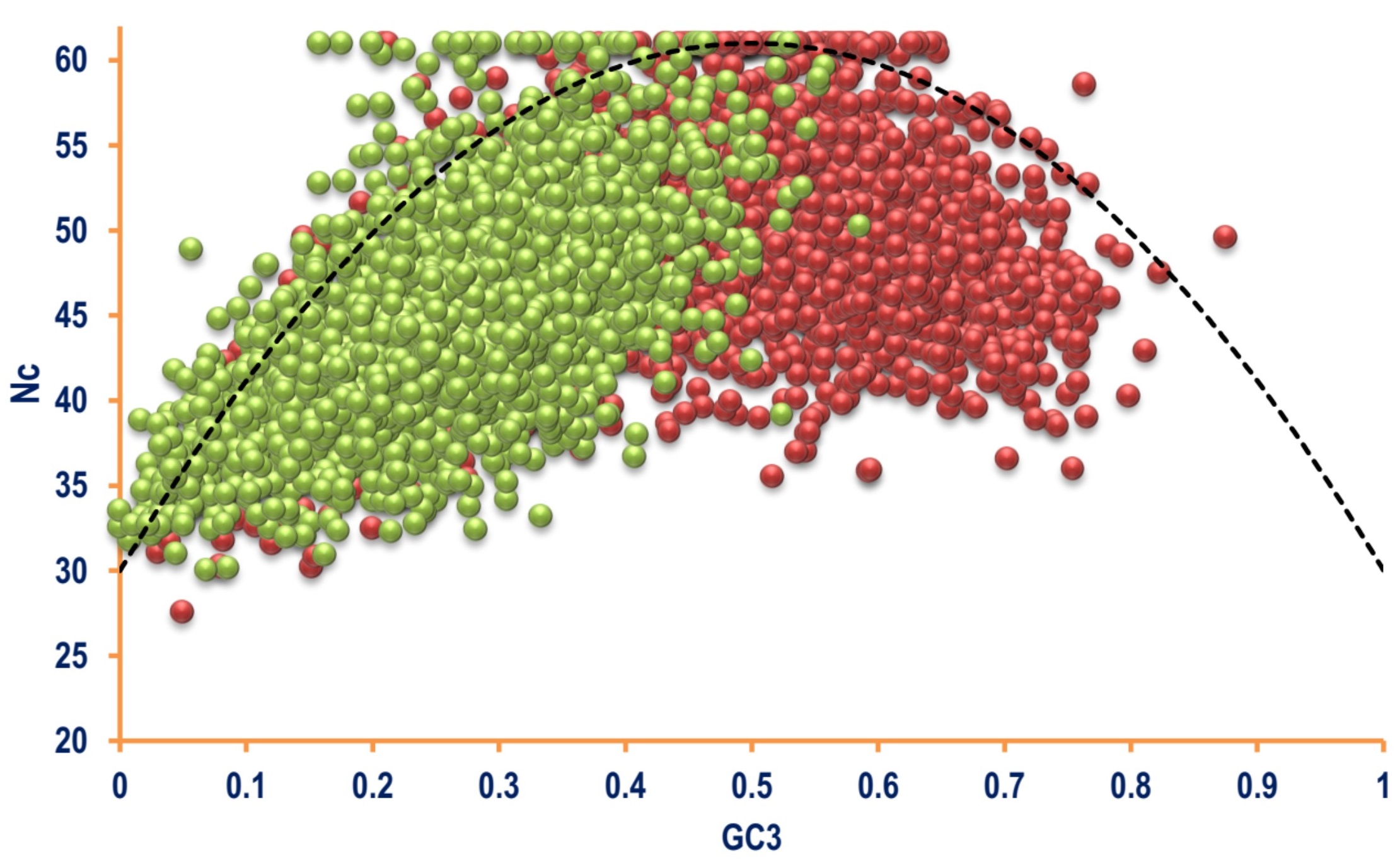

Supplement: Supplementary Figure 1 — A genomic Nc plot demonstrating aberrant mid centric aggregation of coding sequences with right shift in the bacterial species Achromobacter xylosoxidans A8 (shown in red) and Beijerinckia indica indica ATCC 9039 (shown in green). The dashed cyan line represents the null hypothesis curve which suggests that codon usage bias is solely due to mutation and not selection (Wright, 1990). [file Image_1.JPEG]

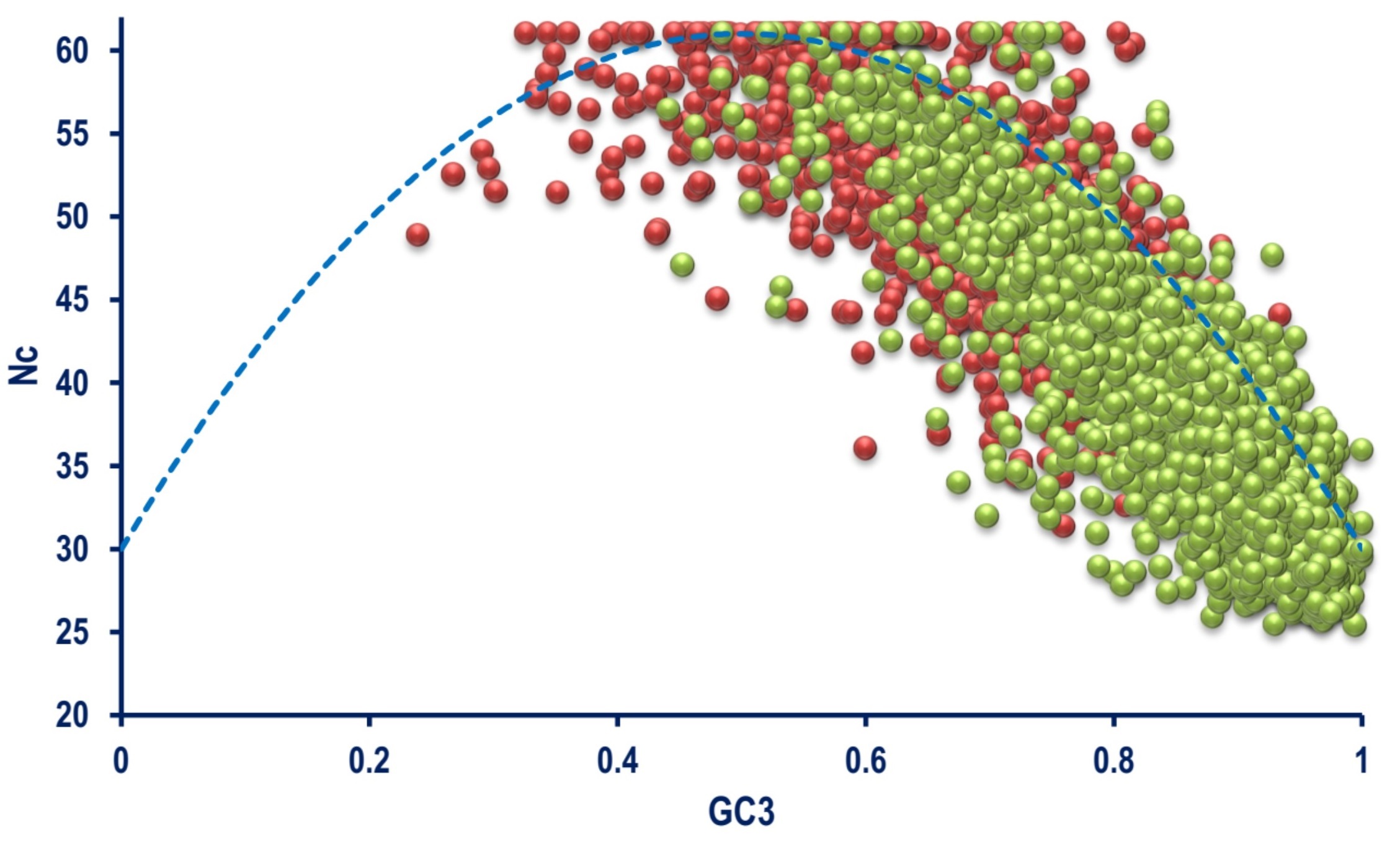

Supplement: Supplementary Figure 2 — A genomic Nc plot demonstrating aberrant inter specific variation in codon usage pattern in the genus Flavobacterium. F. suncheonense (shown in red) demonstrates a mid to left centric aggregation of the coding sequences whereas F. pectinovorum exhibits a left centric aggregation of the coding sequences (shown in green). The dashed black line represents the null hypothesis curve which suggests that codon usage bias is solely due to mutation and not selection (Wright, 1990). [file Image_2.JPEG]
