# Supplementary material for: Comparative Genomic Analysis of Soil Dwelling Bacteria Utilizing a Combinational Codon Usage and Molecular Phylogenetic Approach Accentuating on Key Housekeeping Genes
Source: Front Microbiol. 2019 Dec 17;10:2896. doi: 10.3389/fmicb.2019.02896 (PMC6928123; doi:10.3389/fmicb.2019.02896)
Supplement: Supplementary Table 1 — A detailed list of the 92 soil bacterial species studied along with their NCBI reference sequence number, abbreviated name, Gram nature, habitat and source of isolation. [file Table_1.DOCX]

**Supplementary Table 1: A detailed list of the 92 soil bacterial species studied along with their NCBI reference sequence number, abbreviated name, Gram nature, habitat and source of isolation.**

| Sl. No. | Organism name | NCBI Reference sequence number | Abbreviated name | Gram nature | General Habitat | Source of isolation |
| --- | --- | --- | --- | --- | --- | --- |
| 1 | *Acidocella aminolytica DSM 11237* | NZ_FQVJ00000000.1 | Acami | negative | acid mine | not mentioned |
| 2 | *Acidobacterium capsulatum ATCC 51196* | NC_012483.1 | Accap | negative | acid mine | acid mine drainage |
| 3 | *Acidiphilium cryptum JF-5* | NC_009484.1 | Accry | negative | acid mine | acid mine drainage |
| 4 | *Actinoalloteichus cyanogriseus DSM 43889* | NZ_AUBJ01000001.1 | Accya | positive | soil | soil |
| 5 | *Acidovorax delafieldii 2AN* | NZ_ACQT01000001.1 | Acdel | negative | soil | not mentioned |
| 6 | *Achromobacter denitrificans NBRC 15125* | NZ_BCTQ01000001.1 | Acden | negative | soil | soil |
| 7 | *Acidithiobacillus ferrivorans SS3* | NC_015942.1 | Acfer | negative | acidic soil | not mentioned |
| 8 | *Acidithiobacillus ferrooxidans ATCC 23270* | NC_011761.1 | Acferr | negative | acidic soil | not mentioned |
| 9 | *Acinetobacter calcoaceticus PHEA-2* | NC_016603.1 | Acicalc | negative | soil | not mentioned |
| 10 | *Acidithiobacillus caldus SM-1* | NC_015850.1 | Acicald | negative | acidic soil | not mentioned |
| 11 | *Acidiphilium multivorum AIU301* | NC_015186.1 | Acmul | negative | acid mine | not mentioned |
| 12 | *Acidithiobacillus thiooxidans ATCC 19377* | NZ_AFOH01000122.1 | Acthi | negative | acidic soil | not mentioned |
| 13 | *Achromobacter xylosoxidans A8* | NC_014640.1 | Acxyl | negative | soil | Arsenic-contaminated soil of a pig farm |
| 14 | *Agrobacterium tumefaciens 5A* | NZ_AGVZ01000001.1 | Agtum | negative | soil | agricultural field, USA |
| 15 | *Alcaligenes faecalis P156* | NZ_CP021079.1 | Alfae | negative | soil | surface soil |
| 16 | *Azotobacter chroococcum NCIMB 8003* | NZ_CP010415.1 | Azchr | negative | soil | environmental soil |
| 17 | *Bacillus akibai JCM 9157* | NZ_BAUV01000001.1 | Baaki | positive | soil | not mentioned |
| 18 | *Bacillus atrophaeus 1942* | NC_014639.1 | Baatr | positive | soil | soil |
| 19 | *Bacillus azotoformans LMG 9581* | NZ_AJLR01000146.1 | Baazo | positive | soil | garden soil |
| 20 | *Bacillus circulans NBRC 13626* | NZ_BCVE01000001.1 | Bacir | positive | soil, sewage | not mentioned |
| 21 | *Bacillus clausii KSM-K16* | NC_006582.1 | Bacla | positive | soil, clay | not mentioned |
| 22 | *Bacillus cohnii NBRC 15565* | NZ_BCUW01000001.1 | Bacoh | positive | soil, feces | not mentioned |
| 23 | *Bacillus drentensis NBRC 102427* | NZ_BCUX01000003.1 | Badre | positive | soil | soil in agricultural area |
| 24 | *Bacillus firmus NBRC 15306* | NZ_BCUY01000001.1 | Bafir | positive | soil | not mentioned |
| 25 | *Bacillus flexus Riq5* | NZ_LFQJ01000001.1 | Bafle | positive | soil | not mentioned |
| 26 | *Bacillus horikoshii DSM 8719* | NZ_LQXN01000067.1 | Bahor | positive | soil | not mentioned |
| 27 | *Bacillus krulwichiae NBRC 102362* | NZ_BCVW01000001.1 | Bakru | positive | soil, japan | not mentioned |
| 28 | *Bacillus megaterium WSH-002* | NC_017138.1 | Bameg | positive | soil, cow feces | soil |
| 29 | *Bacillus methanolicus MGA3* | NZ_CP007739.1 | Bamet | positive | soil | soil from marsh |
| 30 | *Bacillus niacini NBRC 15566* | NZ_BCVA01000001.1 | Bania | positive | soil | soil |
| 31 | *Bacillus novalis NBRC 102450* | NZ_BCVP01000009.1 | Banov | positive | soil | argriculture research area |
| 32 | *Bacillus pseudofirmus OF4* | NC_013791.2 | Bapse | positive | soil | alkaline enriched area |
| 33 | *Bacillus pseudomycoides DSM 12442* | NZ_CM000745.1 | Bapseu | positive | soil | soil |
| 34 | *Bacillus pumilus NJ-V2* | NZ_CP012482.1 | Bapum | positive | soil | soil |
| 35 | *Bacillus simplex SH-B26* | NZ_CP011008.1 | Basim | positive | soil | sugar beet rhizosphere |
| 36 | *Bacillus soli NBRC 102451* | NZ_BCVI01000006.1 | Basol | positive | soil | soil |
| 37 | *Bacillus vallismortis DV1-F-3* | NZ_AFSH00000000.1 | Baval | positive | desert soil | soil in death valley, California |
| 38 | *Bacillus vireti LMG 21834* | NZ_ALAN01000059.1 | Bavir | positive | soil | soil |
| 39 | *Bdellovibrio bacteriovorus HD100* | NC_005363.1 | Bdbac | negative | soil | soil |
| 40 | *Beggiatoa alba B18LD* | NZ_JH600070.1 | Bealb | negative | soil | rice field ditch |
| 41 | *Beijerinckia indica indica ATCC 9039* | NC_010581.1 | Beind | negative | soil | acid soil |
| 42 | *Brevibacillus agri BAB-2500* | NZ_AOBR01000008.1 | Bragr | positive | soil | soil |
| 43 | *Burkholderia ambifaria IOP40-10* | NZ_ABLC01000001.1 | Buamb | negative | soil, rhizosphere | rhizosphere |
| 44 | *Burkholderia anthina AZ-4-2-10-S1-D7* | NZ_CM003768.1 | Buant | negative | soil | soil |
| 45 | *Chlorobium phaeovibrioides DSM 265* | NC_009337.1 | Chpha | negative | soil, fresh water | saline intertidal flat |
| 46 | *Chromobacterium subtsugae MWU2387* | NZ_LCWR01000001.1 | Chsub | negative | soil | bog soil |
| 47 | *Chromobacterium vaccinii 21-1* | NZ_CP017707.1 | Chvac | negative | soil | bog soil |
| 48 | *Clostridium acetobutylicum EA 2018* | NC_017295.1 | Clace | positive | soil | soil, china |
| 49 | *Clostridium argentinense CDC 2741* | NZ_AYSO01000020.1 | Clarg | positive | soil | not mentioned |
| 50 | *Clostridium butyricum JKY6D1* | NZ_CP013352.1 | Clbut | positive | mud, soil | pit mud |
| 51 | *Clostridium cadaveris NLAE-zl-G419* | NZ_FOOE01000001.1 | Clcad | positive | soil | not mentioned |
| 52 | *Clostridium cochlearium NLAE-zl-C224* | NZ_FNGL01000001.1 | Clcoc | positive | soil | cow feces enriched soil |
| 53 | *Clostridium pasteurianum DSM 525 = ATCC 6013* | NZ_CP013018.1 | Clpas | positive | soil | soil |
| 54 | *Clostridium scatologenes ATCC 25775* | NZ_CP009933.1 | Clsca | positive | soil | not mentioned |
| 55 | *Clostridium sporogenes NCIMB 10696* | NZ_CP009225.1 | Clspo | positive | soil | soil |
| 56 | *Clostridium tetani 12124569* | NC_022777.1 | Cltet | positive | soil | not mentioned |
| 57 | *Desulfobacterium autotrophicum HRM2, DSM 3382* | NC_012108.1 | Deaut | negative | marine sediment,fresh water | marine sediment |
| 58 | *Desulfobacter postgatei 2ac9* | NZ_CM001488.1 | Depos | negative | marine sediment,brackish water | anaerobic sediment |
| 59 | *Desulfocapsa sulfexigens DSM 10523* | NC_020304.1 | Desul | negative | marine mud, sediment | mud, sediment |
| 60 | *Desulfobacula toluolica Tol2* | NC_018645.1 | Detol | negative | mud | marine mud |
| 61 | *Flavobacterium pectinovorum DSM 6368* | NZ_FRBX01000013.1 | Flpec | negative | soil | soil, england |
| 62 | *Flavobacterium suncheonense GH29-5, DSM 17707* | NZ_KE387020.1 | Flsun | negative | green house soil | green house soil |
| 63 | *Hyphomicrobium denitrificans 1NES1* | NC_021172.1 | Hyden | negative | soil | not mentioned |
| 64 | *Micromonospora aurantiaca ATCC 27029* | NC_014391.1 | Miaur | positive | soil | soil |
| 65 | *Micromonospora carbonacea DSM 43168* | NZ_FMCT01000050.1 | Micar | positive | soil | not mentioned |
| 66 | *Micromonospora chokoriensis DSM 45160* | NZ_LT607409.1 | Micho | positive | sandy soil | not mentioned |
| 67 | *Micromonospora echinospora DSM 43816* | NZ_LT607413.1 | Miech | positive | soil | not mentioned |
| 68 | *Micrococcus luteus NCTC 2665* | NC_012803.1 | Milut | positive | soil | not mentioned |
| 69 | *Micromonospora purpureochromogenes DSM 43821* | NZ_LT607410.1 | Mipur | positive | soil(adobe soil) | not mentioned |
| 70 | *Nitrosomonas communis Nm2* | NZ_CP011451.1 | Nicom | negative | mediterranean soil | not mentioned |
| 71 | *Nitrosomonas europaea ATCC 19718* | NC_004757.1 | Nieur | negative | soil, fresh water | not mentioned |
| 72 | *Nitrobacter hamburgensis X14* | NC_007964.1 | Niham | negative | soil | soil |
| 73 | *Nitrobacter winogradskyi Nb-255* | NC_007406.1 | Niwin | negative | soil | not mentioned |
| 74 | *Nocardia cerradoensis NBRC 101014* | NZ_BAFW01000388.1 | Nocer | positive | soil | not mentioned |
| 75 | *Nocardia otitidiscaviarum IFM 11049* | NZ_BATZ01000065.1 | Nooti | positive | soil | not mentioned |
| 76 | *Pseudomonas azotoformans S4* | NZ_CP014546.1 | Psazo | negative | soil | soil |
| 77 | *Pseudomonas citronellolis P3B5* | NZ_CP014158.1 | Pscit | negative | soil | soil enriched with citronellol |
| 78 | *Pseudomonas fluorescens A506* | NC_017911.1 | Psflu | negative | fresh water,soil,plant | not mentioned |
| 79 | *Pseudomonas mendocina NK-01* | NC_015410.1 | Psmen | negative | fresh water,soil,host | farmland soil |
| 80 | *Pseudomonas oryzihabitans USDA-ARS-USMARC-56511* | NZ_CP013987.1 | Psory | negative | soil | not mentioned |
| 81 | *Pseudomonas putida 1A00316* | NZ_CP014343.1 | Psput | negative | soil | not mentioned |
| 82 | *Rhizobium gallicum IE4872* | NZ_CP017101.1 | Rhgal | negative | soil | nodule from field |
| 83 | *Streptomyces avermitilis MA-4680* | NC_003155.5 | Stave | positive | soil | not mentioned |
| 84 | *Streptomyces clavuligerus ATCC 27064* | NZ_CM001015.1 | Stcla | positive | soil | not mentioned |
| 85 | *Streptomyces hygroscopicus limoneus KCTC 1717* | NZ_CP013219.1 | Sthyg | positive | soil | soil |
| 86 | *Streptomyces noursei ATCC 11455* | NZ_CP011533.1 | Stnou | positive | soil | garden soil |
| 87 | *Streptomyces rubidus CGMCC 4.2026* | NZ_FODD01000110.1 | Strub | positive | soil | not mentioned |
| 88 | *Streptomyces scabrisporus DSM 41855* | NZ_KB889561.1 | Stsca | positive | soil | soil |
| 89 | *Streptomyces vitaminophilus ATCC 31673* | NZ_LLZU01000001.1 | Stvit | positive | soil | soil |
| 90 | *Thiobacillus denitrificans ATCC 25259* | NC_007404.1 | Thden | negative | soil | soil |
| 91 | *Vibrio gazogenes DSM 21264* | NZ_FQUH01000042.1 | Vigaz | negative | soil, marine mud | mud |
| 92 | *Vibrio natriegens NBRC 15636* | NZ_BCUC01000001.1 | Vinat | negative | soil,mud | mud |
