# Supplementary material for: Comparative Genomic Analysis of Soil Dwelling Bacteria Utilizing a Combinational Codon Usage and Molecular Phylogenetic Approach Accentuating on Key Housekeeping Genes
Source: Front Microbiol. 2019 Dec 17;10:2896. doi: 10.3389/fmicb.2019.02896 (PMC6928123; doi:10.3389/fmicb.2019.02896)
Supplement: Supplementary Table 2 — A detailed list showing the genome size (DNA total bases), coding bases, G+C bases, percentage of coding bases and GC content of the genome in the 92 soil bacterial species considered in this study. [file Table_2.DOCX]

**Supplementary Table 2: A detailed list showing the genome size (DNA total bases), coding bases, G+C bases, percentage of coding bases and GC content of the genome in the 92 soil bacterial species considered in this study.**

| Organism name | DNA total bases | Coding bases | G + C bases | Percentage of coding bases | GC content |
| --- | --- | --- | --- | --- | --- |
| Acidocella aminolytica DSM 11237 | 3964820 | 3551814 | 2336168 | 89.58% | 58.92% |
| Acidobacterium capsulatum ATCC 51196 | 4127356 | 3552687 | 2496927 | 86.08% | 60.50% |
| Acidiphilium cryptum JF-5 | 3963080 | 3597137 | 2659039 | 90.77% | 67.10% |
| Actinoalloteichus cyanogriseus DSM 43889 | 6039370 | 5060658 | 4379414 | 83.79% | 72.51% |
| Acidovorax delafieldii 2AN | 4842094 | 4232447 | 3145073 | 87.41% | 64.95% |
| Achromobacter denitrificans NBRC 15125 | 6695638 | 6101426 | 4528489 | 91.13% | 67.63% |
| Acidithiobacillus ferrivorans SS3 | 3207552 | 2913276 | 1815292 | 90.83% | 56.59% |
| Acidithiobacillus ferrooxidans ATCC 23270 | 2982397 | 2681509 | 1752828 | 89.91% | 58.77% |
| Acinetobacter calcoaceticus PHEA-2 | 3862530 | 3389345 | 1499590 | 87.75% | 38.82% |
| Acidithiobacillus caldus SM-1 | 3237599 | 2843068 | 1973499 | 87.81% | 60.96% |
| Acidiphilium multivorum AIU301 | 4214744 | 3832735 | 2822628 | 90.94% | 66.97% |
| Acidithiobacillus thiooxidans ATCC 19377 | 3019868 | 2638807 | 1605449 | 87.38% | 53.16% |
| Achromobacter xylosoxidans A8 | 7359146 | 6729245 | 4840772 | 91.44% | 65.78% |
| Agrobacterium tumefaciens 5A | 5743108 | 5072590 | 3364312 | 88.32% | 58.58% |
| Alcaligenes faecalis P156 | 4026754 | 3612866 | 2281632 | 89.72% | 56.66% |
| Azotobacter chroococcum NCIMB 8003 | 5192286 | 4505533 | 3410631 | 86.77% | 65.69% |
| Bacillus akibai JCM 9157 | 4740369 | 4004114 | 1754938 | 84.47% | 37.02% |
| Bacillus atrophaeus 1942 | 4168266 | 3634724 | 1801495 | 87.20% | 43.22% |
| Bacillus azotoformans LMG 9581 | 4223247 | 3622951 | 1572659 | 85.79% | 37.24% |
| Bacillus circulans NBRC 13626 | 5095903 | 4272559 | 1812459 | 83.84% | 35.57% |
| Bacillus clausii KSM-K16 | 4303871 | 3747396 | 1926125 | 87.07% | 44.75% |
| Bacillus cohnii NBRC 15565 | 4893042 | 4196979 | 1745465 | 85.77% | 35.67% |
| Bacillus drentensis NBRC 102427 | 5151471 | 4391488 | 1993500 | 85.25% | 38.70% |
| Bacillus firmus NBRC 15306 | 4402977 | 3768128 | 1835070 | 85.58% | 41.68% |
| Bacillus flexus Riq5 | 5639405 | 4717668 | 2127826 | 83.66% | 37.73% |
| Bacillus horikoshii DSM 8719 | 4674763 | 3970942 | 1931928 | 84.94% | 41.33% |
| Bacillus krulwichiae NBRC 102362 | 4516276 | 3919128 | 1713241 | 86.78% | 37.93% |
| Bacillus megaterium WSH-002 | 5075293 | 4296350 | 1938135 | 84.65% | 38.19% |
| Bacillus methanolicus MGA3 | 3425208 | 2846442 | 1321767 | 83.10% | 38.59% |
| Bacillus niacini NBRC 15566 | 6181012 | 5326702 | 2358498 | 86.18% | 38.16% |
| Bacillus novalis NBRC 102450 | 5566063 | 4729894 | 2218436 | 84.98% | 39.86% |
| Bacillus pseudofirmus OF4 | 4249248 | 3641666 | 1693774 | 85.70% | 39.86% |
| Bacillus pseudomycoides DSM 12442 | 5752014 | 4484526 | 2034410 | 77.96% | 35.37% |
| Bacillus pumilus NJ-V2 | 3787818 | 3374140 | 1563670 | 89.08% | 41.28% |
| Bacillus simplex SH-B26 | 5521719 | 4422034 | 2195482 | 80.08% | 39.76% |
| Bacillus soli NBRC 102451 | 5459242 | 4612662 | 2158311 | 84.49% | 39.53% |
| Bacillus vallismortis DV1-F-3 | 3871829 | 3428546 | 1694340 | 88.55% | 43.76% |
| Bacillus vireti LMG 21834 | 5283728 | 4466160 | 2099808 | 84.53% | 39.74% |
| Bdellovibrio bacteriovorus HD100 | 3782950 | 3524879 | 1915894 | 93.18% | 50.65% |
| Beggiatoa alba B18LD | 4265146 | 3723359 | 1736797 | 87.30% | 40.72% |
| Beijerinckia indica indica ATCC 9039 | 4418616 | 3652924 | 2517858 | 82.67% | 56.98% |
| Brevibacillus agri BAB-2500 | 5386487 | 4457110 | 2884144 | 82.75% | 53.54% |
| Burkholderia ambifaria IOP40-10 | 7689995 | 6353567 | 5101781 | 82.62% | 66.34% |
| Burkholderia anthina AZ-4-2-10-S1-D7 | 4513863 | 3962088 | 3035011 | 87.78% | 67.24% |
| Chlorobium phaeovibrioides DSM 265 | 1966858 | 1814201 | 1042272 | 92.24% | 52.99% |
| Chromobacterium subtsugae MWU2387 | 5100973 | 4534834 | 3276988 | 88.90% | 64.24% |
| Chromobacterium vaccinii 21-1 | 5041230 | 4497624 | 3240118 | 89.22% | 64.27% |
| Clostridium acetobutylicum EA 2018 | 4132226 | 3627063 | 1277909 | 87.78% | 30.93% |
| Clostridium argentinense CDC 2741 | 4742562 | 3760582 | 1356552 | 79.29% | 28.60% |
| Clostridium butyricum JKY6D1 | 4618327 | 3936717 | 1327045 | 85.24% | 28.73% |
| Clostridium cadaveris NLAE-zl-G419 | 3532192 | 3114145 | 1097873 | 88.16% | 31.08% |
| Clostridium cochlearium NLAE-zl-C224 | 2400722 | 2113537 | 678307 | 88.04% | 28.25% |
| Clostridium pasteurianum DSM 525 = ATCC 6013 | 4352101 | 3642265 | 1302869 | 83.69% | 29.94% |
| Clostridium scatologenes ATCC 25775 | 7353834 | 6267862 | 2228285 | 85.23% | 30.30% |
| Clostridium sporogenes NCIMB 10696 | 4141984 | 3449935 | 1159032 | 83.29% | 27.98% |
| Clostridium tetani 12124569 | 2807481 | 2479009 | 808919 | 88.30% | 28.81% |
| Desulfobacterium autotrophicum HRM2, DSM 3382 | 5657782 | 5019916 | 2758941 | 88.73% | 48.76% |
| Desulfobacter postgatei 2ac9 | 3974658 | 3556920 | 1874851 | 89.49% | 47.17% |
| Desulfocapsa sulfexigens DSM 10523 | 4023512 | 3618439 | 1825701 | 89.93% | 45.38% |
| Desulfobacula toluolica Tol2 | 5197905 | 4564864 | 2154472 | 87.82% | 41.45% |
| Flavobacterium pectinovorum DSM 6368 | 5610979 | 4983727 | 1882665 | 88.82% | 33.55% |
| Flavobacterium suncheonense GH29-5, DSM 17707 | 2880663 | 2622751 | 1165575 | 91.05% | 40.46% |
| Hyphomicrobium denitrificans 1NES1 | 3808687 | 3336523 | 2273818 | 87.60% | 59.70% |
| Micromonospora aurantiaca ATCC 27029 | 7025559 | 6363154 | 5116162 | 90.57% | 72.82% |
| Micromonospora carbonacea DSM 43168 | 7941928 | 7078216 | 5854247 | 89.12% | 73.71% |
| Micromonospora chokoriensis DSM 45160 | 6897719 | 6188562 | 4928934 | 89.72% | 71.46% |
| Micromonospora echinospora DSM 43816 | 7775486 | 6967622 | 5622538 | 89.61% | 72.31% |
| Micrococcus luteus NCTC 2665 | 2501097 | 2282638 | 1825682 | 91.27% | 73.00% |
| Micromonospora purpureochromogenes DSM 43821 | 6674134 | 5937595 | 4870943 | 88.96% | 72.98% |
| Nitrosomonas communis Nm2 | 4067838 | 3269523 | 1819617 | 80.37% | 44.73% |
| Nitrosomonas europaea ATCC 19718 | 2812094 | 2489540 | 1426243 | 88.53% | 50.72% |
| Nitrobacter hamburgensis X14 | 5011522 | 4207807 | 3087965 | 83.96% | 61.62% |
| Nitrobacter winogradskyi Nb-255 | 3402093 | 2891362 | 2110965 | 84.99% | 62.05% |
| Nocardia cerradoensis NBRC 101014 | 7602329 | 6830893 | 5181529 | 89.85% | 68.16% |
| Nocardia otitidiscaviarum IFM 11049 | 7895563 | 7065235 | 5438668 | 89.48% | 68.88% |
| Pseudomonas azotoformans S4 | 6859618 | 6217649 | 4137302 | 90.64% | 60.31% |
| Pseudomonas citronellolis P3B5 | 6951444 | 6042133 | 4665300 | 86.92% | 67.11% |
| Pseudomonas fluorescens A506 | 6019547 | 5363601 | 3609012 | 89.10% | 59.95% |
| Pseudomonas mendocina NK-01 | 5434353 | 4838314 | 3396902 | 89.03% | 62.51% |
| Pseudomonas oryzihabitans USDA-ARS-USMARC-56511 | 4834356 | 4337371 | 3147806 | 89.72% | 65.11% |
| Pseudomonas putida 1A00316 | 5715815 | 5131574 | 3683209 | 89.78% | 64.44% |
| Rhizobium gallicum IE4872 | 7474202 | 6448084 | 4456959 | 86.27% | 59.63% |
| Streptomyces avermitilis MA-4680 | 9119895 | 7883435 | 6447889 | 86.44% | 70.70% |
| Streptomyces clavuligerus ATCC 27064 | 9134976 | 7572895 | 6612700 | 82.90% | 72.39% |
| Streptomyces hygroscopicus limoneus KCTC 1717 | 10537932 | 9123060 | 7583141 | 86.57% | 71.96% |
| Streptomyces noursei ATCC 11455 | 9815884 | 8388269 | 7013746 | 85.46% | 71.45% |
| Streptomyces rubidus CGMCC 4.2026 | 9005705 | 7839180 | 6566269 | 87.05% | 72.91% |
| Streptomyces scabrisporus DSM 41855 | 11392659 | 9674153 | 8079877 | 84.92% | 70.92% |
| Streptomyces vitaminophilus ATCC 31673 | 6549812 | 5641328 | 4716565 | 86.13% | 72.01% |
| Thiobacillus denitrificans ATCC 25259 | 2909809 | 2700629 | 1922430 | 92.81% | 66.07% |
| Vibrio gazogenes DSM 21264 | 4635841 | 3989501 | 2089844 | 86.06% | 45.08% |
| Vibrio natriegens NBRC 15636 | 5096560 | 4440061 | 2293441 | 87.12% | 45.00% |
