# Supplementary material for: Comparative Genomic Analysis of Soil Dwelling Bacteria Utilizing a Combinational Codon Usage and Molecular Phylogenetic Approach Accentuating on Key Housekeeping Genes
Source: Front Microbiol. 2019 Dec 17;10:2896. doi: 10.3389/fmicb.2019.02896 (PMC6928123; doi:10.3389/fmicb.2019.02896)
Supplement: Supplementary Table 3 — Results of the correlation analysis between the different codon usage parameters at the whole genome level in the 92 soil bacterial species considered in this study. [file Table_3.DOCX]

**Supplementary Table 3: Results of the correlation analysis between the different codon usage parameters at the whole genome level in the 92 soil bacterial species considered in this study.**

| Total organisms | Spearman rank order correlation coefficient (p<0.01) | | | | | |
| --- | --- | --- | --- | --- | --- | --- |
|  | **Nc and GC3** | **Nc and length** | **Nc and hydrophobicity** | **GC3 and length** | **GC3 and hydrophobicity** | **Length and hydrophobicity** |
| *Acidocella aminolytica DSM 11237* | -0.79 | -0.13 | -0.01 | 0.09 | 0.07 | 0.03 |
| *Acidobacterium capsulatum ATCC 51196* | -0.87 | -0.07 | -0.01 | 0.03 | 0.05 | -0.05 |
| *Acidiphilium cryptum JF-5* | -0.88 | -0.09 | -0.09 | 0.09 | 0.12 | 0.00 |
| *Actinoalloteichus cyanogriseus DSM 43889* | -0.79 | -0.15 | -0.15 | 0.12 | 0.25 | 0.00 |
| *Acidovorax delafieldii 2AN* | -0.86 | -0.10 | -0.04 | 0.07 | 0.12 | -0.02 |
| *Achromobacter denitrificans NBRC 15125* | -0.81 | -0.17 | -0.05 | 0.14 | 0.11 | -0.03 |
| *Acidithiobacillus ferrivorans SS3* | -0.70 | -0.18 | 0.02 | 0.17 | 0.01 | -0.05 |
| *Acidithiobacillus ferrooxidans ATCC 23270* | -0.73 | -0.17 | -0.01 | 0.16 | 0.03 | -0.01 |
| *Acinetobacter calcoaceticus PHEA-2* | 0.64 | -0.12 | 0.11 | 0.00 | 0.18 | -0.03 |
| *Acidithiobacillus caldus SM-1* | -0.80 | -0.12 | 0.01 | 0.11 | 0.00 | -0.01 |
| *Acidiphilium multivorum AIU301* | -0.90 | -0.12 | -0.15 | 0.13 | 0.18 | 0.00 |
| *Acidithiobacillus thiooxidans ATCC 19377* | -0.57 | -0.12 | -0.03 | 0.10 | 0.03 | -0.03 |
| *Achromobacter xylosoxidans A8* | -0.86 | -0.13 | -0.06 | 0.10 | 0.11 | -0.03 |
| *Agrobacterium tumefaciens 5A* | -0.66 | -0.18 | -0.01 | 0.15 | 0.10 | -0.02 |
| *Alcaligenes faecalis P156* | -0.48 | -0.15 | -0.03 | 0.09 | 0.17 | -0.02 |
| *Azotobacter chroococcum NCIMB 8003* | -0.88 | -0.18 | -0.13 | 0.15 | 0.17 | -0.03 |
| *Bacillus akibai JCM 9157* | 0.59 | -0.07 | 0.02 | 0.01 | 0.10 | -0.03 |
| *Bacillus atrophaeus 1942* | 0.42 | 0.03 | 0.05 | 0.11 | 0.15 | -0.02 |
| *Bacillus azotoformans LMG 9581* | 0.63 | -0.03 | 0.01 | 0.06 | 0.06 | -0.02 |
| *Bacillus circulans NBRC 13626* | 0.55 | -0.05 | 0.07 | 0.00 | 0.04 | -0.01 |
| *Bacillus clausii KSM-K16* | 0.21 | -0.03 | 0.04 | 0.14 | 0.11 | 0.02 |
| *Bacillus cohnii NBRC 15565* | 0.55 | -0.06 | 0.02 | -0.02 | 0.04 | -0.02 |
| *Bacillus drentensis NBRC 102427* | 0.56 | 0.02 | 0.01 | 0.07 | 0.03 | -0.04 |
| *Bacillus firmus NBRC 15306* | 0.45 | 0.00 | 0.04 | 0.05 | 0.06 | 0.00 |
| *Bacillus flexus Riq5* | 0.58 | -0.03 | 0.09 | 0.03 | 0.09 | 0.04 |
| *Bacillus horikoshii DSM 8719* | 0.48 | 0.02 | 0.07 | 0.04 | 0.08 | -0.02 |
| *Bacillus krulwichiae NBRC 102362* | 0.62 | -0.08 | 0.04 | 0.00 | 0.13 | 0.00 |
| *Bacillus megaterium WSH-002* | 0.57 | -0.04 | 0.09 | 0.01 | 0.09 | 0.02 |
| *Bacillus methanolicus MGA3* | 0.53 | -0.02 | 0.00 | 0.07 | -0.02 | -0.03 |
| *Bacillus niacini NBRC 15566* | 0.60 | 0.00 | 0.02 | 0.05 | 0.05 | -0.04 |
| *Bacillus novalis NBRC 102450* | 0.52 | 0.05 | 0.02 | 0.10 | 0.07 | -0.04 |
| *Bacillus pseudofirmus OF4* | 0.61 | -0.05 | 0.05 | -0.01 | 0.11 | -0.02 |
| *Bacillus pseudomycoides DSM 12442* | 0.56 | -0.05 | 0.04 | 0.03 | 0.04 | 0.01 |
| *Bacillus pumilus NJ-V2* | 0.53 | -0.03 | 0.06 | 0.03 | 0.19 | 0.00 |
| *Bacillus simplex SH-B26* | 0.60 | 0.07 | 0.05 | 0.09 | 0.03 | 0.00 |
| *Bacillus soli NBRC 102451* | 0.55 | 0.05 | 0.04 | 0.11 | 0.10 | -0.04 |
| *Bacillus vallismortis DV1-F-3* | 0.31 | 0.01 | 0.01 | 0.11 | 0.18 | 0.00 |
| *Bacillus vireti LMG 21834* | 0.50 | 0.04 | 0.04 | 0.11 | 0.07 | -0.02 |
| *Bdellovibrio bacteriovorus HD100* | 0.01 | -0.13 | 0.05 | 0.07 | 0.03 | -0.02 |
| *Beggiatoa alba B18LD* | 0.12 | -0.04 | -0.02 | 0.14 | 0.04 | -0.05 |
| *Beijerinckia indica indica ATCC 9039* | -0.78 | -0.16 | -0.02 | 0.13 | 0.03 | 0.04 |
| *Brevibacillus agri BAB-2500* | -0.52 | -0.22 | -0.09 | 0.17 | 0.19 | -0.02 |
| *Burkholderia ambifaria IOP40-10* | -0.87 | -0.16 | -0.16 | 0.13 | 0.22 | 0.01 |
| *Burkholderia anthina AZ-4-2-10-S1-D7* | -0.83 | -0.17 | -0.16 | 0.13 | 0.22 | 0.02 |
| *Chlorobium phaeovibrioides DSM 265* | -0.45 | -0.06 | -0.02 | 0.08 | 0.00 | -0.02 |
| *Chromobacterium subtsugae MWU2387* | -0.81 | -0.11 | -0.08 | 0.08 | 0.13 | -0.03 |
| *Chromobacterium vaccinii 21-1* | -0.77 | -0.16 | -0.09 | 0.12 | 0.12 | -0.03 |
| *Clostridium acetobutylicum EA 2018* | 0.55 | -0.07 | 0.08 | -0.07 | 0.05 | -0.03 |
| *Clostridium argentinense CDC 2741* | 0.62 | -0.09 | 0.03 | -0.09 | 0.00 | -0.03 |
| *Clostridium butyricum JKY6D1* | 0.54 | -0.12 | -0.02 | -0.14 | -0.03 | -0.06 |
| *Clostridium cadaveris NLAE-zl-G419* | 0.63 | -0.08 | 0.00 | -0.10 | 0.00 | -0.05 |
| *Clostridium cochlearium NLAE-zl-C224* | 0.62 | -0.09 | -0.01 | -0.08 | -0.06 | -0.07 |
| *Clostridium pasteurianum DSM 525 = ATCC 6013* | 0.58 | -0.03 | 0.07 | 0.00 | 0.05 | -0.04 |
| *Clostridium scatologenes ATCC 25775* | 0.61 | -0.10 | 0.01 | -0.09 | -0.04 | 0.00 |
| *Clostridium sporogenes NCIMB 10696* | 0.58 | -0.08 | 0.06 | -0.06 | 0.05 | -0.03 |
| *Clostridium tetani 12124569* | 0.53 | -0.12 | 0.07 | -0.08 | 0.04 | -0.06 |
| *Desulfobacterium autotrophicum HRM2, DSM 3382* | -0.42 | -0.14 | -0.16 | 0.15 | 0.19 | -0.04 |
| *Desulfobacter postgatei 2ac9* | -0.44 | -0.17 | -0.11 | 0.22 | 0.15 | -0.02 |
| *Desulfocapsa sulfexigens DSM 10523* | 0.17 | -0.05 | -0.05 | 0.10 | 0.05 | -0.04 |
| *Desulfobacula toluolica Tol2* | 0.14 | -0.05 | -0.08 | 0.15 | 0.06 | -0.01 |
| *Flavobacterium pectinovorum DSM 6368* | 0.66 | -0.08 | 0.00 | -0.01 | -0.03 | -0.06 |
| *Flavobacterium suncheonense GH29-5, DSM 17707* | 0.32 | -0.07 | 0.06 | 0.06 | 0.07 | -0.04 |
| *Hyphomicrobium denitrificans 1NES1* | -0.77 | -0.21 | 0.04 | 0.17 | -0.01 | 0.00 |
| *Micromonospora aurantiaca ATCC 27029* | -0.86 | -0.09 | -0.13 | 0.08 | 0.22 | 0.01 |
| *Micromonospora carbonacea DSM 43168* | -0.86 | -0.10 | -0.17 | 0.11 | 0.26 | 0.01 |
| *Micromonospora chokoriensis DSM 45160* | -0.80 | -0.13 | -0.07 | 0.11 | 0.18 | 0.00 |
| *Micromonospora echinospora DSM 43816* | -0.85 | -0.10 | -0.11 | 0.09 | 0.20 | -0.01 |
| *Micrococcus luteus NCTC 2665* | -0.86 | -0.16 | -0.10 | 0.14 | 0.21 | -0.01 |
| *Micromonospora purpureochromogenes DSM 43821* | -0.87 | -0.12 | -0.11 | 0.10 | 0.19 | 0.00 |
| *Nitrosomonas communis Nm2* | 0.07 | -0.01 | 0.04 | 0.15 | -0.01 | 0.02 |
| *Nitrosomonas europaea ATCC 19718* | -0.46 | -0.16 | 0.00 | 0.17 | 0.01 | -0.04 |
| *Nitrobacter hamburgensis X14* | -0.85 | -0.16 | -0.10 | 0.17 | 0.13 | 0.01 |
| *Nitrobacter winogradskyi Nb-255* | -0.83 | -0.20 | -0.11 | 0.21 | 0.15 | 0.00 |
| *Nocardia cerradoensis NBRC 101014* | -0.71 | -0.08 | -0.08 | 0.08 | 0.13 | 0.01 |
| *Nocardia otitidiscaviarum IFM 11049* | -0.76 | -0.07 | -0.09 | 0.09 | 0.20 | 0.01 |
| *Pseudomonas azotoformans S4* | -0.72 | -0.15 | -0.05 | 0.12 | 0.17 | -0.04 |
| *Pseudomonas citronellolis P3B5* | -0.89 | -0.14 | -0.09 | 0.11 | 0.13 | -0.02 |
| *Pseudomonas fluorescens A506* | -0.69 | -0.14 | -0.07 | 0.12 | 0.20 | -0.05 |
| *Pseudomonas mendocina NK-01* | -0.76 | -0.16 | -0.04 | 0.13 | 0.15 | -0.03 |
| *Pseudomonas oryzihabitans USDA-ARS-USMARC-56511* | -0.73 | -0.15 | -0.06 | 0.12 | 0.17 | -0.02 |
| *Pseudomonas putida 1A00316* | -0.81 | -0.13 | -0.07 | 0.10 | 0.15 | -0.02 |
| *Rhizobium gallicum IE4872* | -0.81 | -0.15 | -0.03 | 0.12 | 0.06 | 0.01 |
| *Streptomyces avermitilis MA-4680* | -0.82 | -0.14 | -0.15 | 0.12 | 0.23 | 0.01 |
| *Streptomyces clavuligerus ATCC 27064* | -0.79 | -0.13 | -0.11 | 0.14 | 0.22 | 0.00 |
| *Streptomyces hygroscopicus limoneus KCTC 1717* | -0.85 | -0.15 | -0.19 | 0.14 | 0.24 | 0.02 |
| *Streptomyces noursei ATCC 11455* | -0.86 | -0.12 | -0.15 | 0.12 | 0.23 | 0.04 |
| *Streptomyces rubidus CGMCC 4.2026* | -0.86 | -0.16 | -0.15 | 0.15 | 0.21 | -0.01 |
| *Streptomyces scabrisporus DSM 41855* | -0.79 | -0.14 | -0.18 | 0.14 | 0.28 | 0.04 |
| *Streptomyces vitaminophilus ATCC 31673* | -0.82 | -0.16 | -0.15 | 0.14 | 0.23 | -0.03 |
| *Thiobacillus denitrificans ATCC 25259* | -0.83 | -0.16 | -0.04 | 0.13 | 0.14 | -0.02 |
| *Vibrio gazogenes DSM 21264* | 0.26 | -0.08 | 0.00 | 0.16 | 0.12 | -0.03 |
| *Vibrio natriegens NBRC 15636* | 0.62 | -0.14 | 0.06 | 0.02 | 0.11 | -0.02 |
