# Supplementary material for: Comparative Genomic Analysis of Soil Dwelling Bacteria Utilizing a Combinational Codon Usage and Molecular Phylogenetic Approach Accentuating on Key Housekeeping Genes
Source: Front Microbiol. 2019 Dec 17;10:2896. doi: 10.3389/fmicb.2019.02896 (PMC6928123; doi:10.3389/fmicb.2019.02896)
Supplement: Supplementary Table 4 — A list showing the genomic gene count along with the percentage (%age) of genes in the genome of the 92 soil bacterial species considered in this study that is below the genic Nc value of the housekeeping genes rpoB, atpD, infB, and trpB. [file Table_4.DOCX]

**Supplementary Table 4: A list showing the genomic gene count along with the percentage (%age) of genes in the genome of the 92 soil bacterial species considered in this study that is below the genic Nc value of the housekeeping genes *rpoB*, *atpD*, *infB*, and *trpB*.**

| **Organism name** | **Abbreviated name** | **Total Genomic Gene Count** | ***%age of genes in genome having Nc less than rpoB*** | ***%age of genes in genome having Nc less than atpD*** | ***%age of genes in genome having Nc less than infB*** | ***%age of genes in genome having Nc less than trpB*** |
| --- | --- | --- | --- | --- | --- | --- |
| *Achromobacter denitrificans NBRC 15125* | Acden | 5841 | 18.80 | 8.01 | 16.69 | 27.31 |
| *Achromobacter xylosoxidans A8* | Acxyl | 6417 | 12.81 | 9.47 | 7.36 | 12.98 |
| *Acidiphilium cryptum JF-5* | Accry | 2919 | 5.62 | 1.34 | 63.48 | 7.47 |
| *Acidiphilium multivorum AIU301* | Acmul | 3607 | 3.77 | 0.86 | 55.86 | 5.52 |
| *Acidithiobacillus caldus SM-1* | Acicald | 2787 | 13.99 | 17.19 | 17.62 | 58.34 |
| *Acidithiobacillus ferrivorans SS3* | Acfer | 2652 | 57.35 | 5.69 | 54.52 | 86.88 |
| *Acidithiobacillus ferrooxidans ATCC 23270* | Acferr | 2584 | 37.58 | 32.08 | 38.20 | 43.19 |
| *Acidithiobacillus thiooxidans ATCC 19377* | Acthi | 2579 | 28.54 | 9.93 | 37.65 | 31.33 |
| *Acidobacterium capsulatum ATCC 51196* | Accap | 3073 | 7.71 | 8.04 | 36.02 | 32.22 |
| *Acidocella aminolytica DSM 11237* | Acami | 3391 | 5.75 | 1.30 | 5.28 | 11.12 |
| *Acidovorax delafieldii 2AN* | Acdel | 3982 | 25.94 | 28.50 | 41.59 | 26.07 |
| *Acinetobacter calcoaceticus PHEA-2* | Acicalc | 3291 | 5.07 | 2.49 | 2.89 | 9.45 |
| *Actinoalloteichus cyanogriseus DSM 43889* | Accya | 4462 | 8.02 | 2.11 | 36.73 | 16.36 |
| *Agrobacterium tumefaciens 5A* | Agtum | 4946 | 2.22 | 0.57 | 7.84 | 16.96 |
| *Alcaligenes faecalis P156* | Alfae | 3450 | 9.71 | 2.67 | 18.43 | 51.57 |
| *Azotobacter chroococcum NCIMB 8003* | Azchr | 4132 | 50.24 | 6.53 | 56.29 | 3.82 |
| *Bacillus akibai JCM 9157* | Baaki | 3707 | 6.74 |  | 18.37 | 72.43 |
| *Bacillus atrophaeus 1942* | Baatr | 3468 | 13.90 | 6.46 | 20.96 | 66.58 |
| *Bacillus azotoformans LMG 9581* | Baazo | 3592 | 27.53 | 3.15 | 10.08 | 26.73 |
| *Bacillus circulans NBRC 13626* | Bacir | 4274 | 9.17 | 9.76 | 9.71 | 32.41 |
| *Bacillus clausii KSM-K16* | Bacla | 3769 | 30.54 | 22.63 | 13.61 | 55.98 |
| *Bacillus cohnii NBRC 15565* | Bacoh | 4223 | 25.46 | 3.05 | 7.70 | 69.43 |
| *Bacillus drentensis NBRC 102427* | Badre | 4272 | 14.40 | 5.27 | 22.07 | 25.77 |
| *Bacillus firmus NBRC 15306* | Bafir | 3689 | 14.50 | 4.09 | 12.85 | 47.66 |
| *Bacillus flexus Riq5* | Bafle | 4845 | 3.92 | 1.90 | 6.46 | 46.58 |
| *Bacillus horikoshii DSM 8719* | Bahor | 3978 | 13.78 | 1.18 | 5.46 | 78.86 |
| *Bacillus krulwichiae NBRC 102362* | Bakru | 3864 | 12.55 | 5.43 | 4.84 | 60.46 |
| *Bacillus megaterium WSH-002* | Bameg | 4244 | 3.56 | 1.93 | 4.88 | 29.52 |
| *Bacillus methanolicus MGA3* | Bamet | 2719 | 28.10 | 8.46 | 25.41 | 45.16 |
| *Bacillus niacini NBRC 15566* | Bania | 5150 | 7.17 | 2.68 | 8.74 | 62.47 |
| *Bacillus novalis NBRC 102450* | Banov | 4636 | 19.76 | 6.04 | 20.62 | 36.95 |
| *Bacillus pseudofirmus OF4* | Bapse | 3612 | 7.92 | 1.19 | 11.02 | 38.73 |
| *Bacillus pseudomycoides DSM 12442* | Bapseu | 4714 | 15.53 | 3.46 | 14.64 | 53.10 |
| *Bacillus pumilus NJ-V2* | Bapum | 3323 | 14.78 | 2.74 | 18.45 | 64.43 |
| *Bacillus simplex SH-B26* | Basim | 4214 | 11.77 | 1.97 | 10.37 | 73.45 |
| *Bacillus soli NBRC 102451* | Basol | 4498 | 30.04 | 8.51 | 15.81 | 13.41 |
| *Bacillus vallismortis DV1-F-3* | Baval | 3142 |  | 4.68 | 31.09 | 69.19 |
| *Bacillus vireti LMG 21834* | Bavir | 4417 | 16.28 | 6.72 | 26.42 | 45.23 |
| *Bdellovibrio bacteriovorus HD100* | Bdbac | 3348 | 2.39 | 0.54 | 8.21 |  |
| *Beggiatoa alba B18LD* | Bealb | 3197 | 75.07 | 6.57 | 76.63 | 65.59 |
| *Beijerinckia indica indica ATCC 9039* | Beind | 3295 | 7.98 | 2.82 | 20.58 | 39.21 |
| *Brevibacillus agri BAB-2500* | Bragr | 4398 | 43.47 | 6.57 | 48.34 | 43.70 |
| *Burkholderia ambifaria IOP40-10* | Buamb | 6128 | 15.71 | 7.46 | 7.80 | 9.12 |
| *Burkholderia anthina AZ-4-2-10-S1-D7* | Buant | 5902 | 8.66 | 9.45 | 13.17 | 10.66 |
| *Chlorobium phaeovibrioides DSM 265* | Chpha | 1661 | 40.76 | 37.03 | 33.84 | 28.24 |
| *Chromobacterium subtsugae MWU2387* | Chsub | 4387 | 7.29 | 27.81 | 9.10 | 31.37 |
| *Chromobacterium vaccinii 21-1* | Chvac | 4284 | 14.47 | 34.15 | 32.38 | 44.89 |
| *Clostridium acetobutylicum EA 2018* | Clace | 3440 | 33.43 | 1.54 | 16.83 | 15.96 |
| *Clostridium argentinense CDC 2741* | Clarg | 3685 | 57.94 | 83.64 | 14.95 |  |
| *Clostridium butyricum JKY6D1* | Clbut | 3653 | 36.98 | 11.20 | 16.18 | 76.43 |
| *Clostridium cadaveris NLAE-zl-G419* | Clcad | 2935 | 46.34 | 4.87 | 5.21 |  |
| *Clostridium cochlearium NLAE-zl-C224* | Clcoc | 2012 | 53.13 | 42.35 | 34.69 |  |
| *Clostridium pasteurianum DSM 525 = ATCC 6013* | Clpas | 3464 | 29.39 | 18.68 | 10.71 | 13.45 |
| *Clostridium scatologenes ATCC 25775* | Clsca | 4618 | 24.97 | 12.95 | 10.78 | 10.37 |
| *Clostridium sporogenes NCIMB 10696* | Clspo | 3287 | 36.23 | 16.88 | 29.15 |  |
| *Clostridium tetani 12124569* | Cltet | 2346 | 41.30 |  | 7.76 |  |
| *Desulfobacter postgatei 2ac9* | Depos | 3104 | 42.78 | 6.57 | 34.70 | 1.32 |
| *Desulfobacterium autotrophicum HRM2, DSM 3382* | Deaut | 4355 | 44.68 | 15.20 | 74.14 | 68.70 |
| *Desulfobacula toluolica Tol2* | Detol | 4053 | 49.07 | 0.27 | 38.27 | 42.29 |
| *Desulfocapsa sulfexigens DSM 10523* | Desul | 3182 | 8.52 | 70.71 | 9.59 | 24.70 |
| *Flavobacterium pectinovorum DSM 6368* | Flpec | 4327 | 10.42 | 5.38 | 14.35 | 60.48 |
| *Flavobacterium suncheonense GH29-5, DSM 17707* | Flsun | 2495 | 16.87 | 8.02 | 36.67 |  |
| *Hyphomicrobium denitrificans 1NES1* | Hyden | 3181 | 0.47 | 0.50 | 20.94 | 32.03 |
| *Micrococcus luteus NCTC 2665* | Milut | 2114 | 5.91 | 1.99 | 23.23 | 34.96 |
| *Micromonospora aurantiaca ATCC 27029* | Miaur | 5823 | 19.66 | 3.50 | 72.57 | 3.92 |
| *Micromonospora carbonacea DSM 43168* | Micar | 6216 | 30.69 | 11.42 | 72.55 | 28.78 |
| *Micromonospora chokoriensis DSM 45160* | Micho | 5628 | 18.51 | 6.15 | 33.14 | 6.66 |
| *Micromonospora echinospora DSM 43816* | Miech | 5962 | 8.81 | 2.03 | 49.80 | 2.38 |
| *Micromonospora purpureochromogenes DSM 43821* | Mipur | 5531 | 38.55 | 9.47 | 73.19 | 4.05 |
| *Nitrobacter hamburgensis X14* | Niham | 3410 | 0.44 | 1.70 | 1.14 | 8.71 |
| *Nitrobacter winogradskyi Nb-255* | Niwin | 2625 | 2.06 | 1.75 | 0.91 | 6.06 |
| *Nitrosomonas communis Nm2* | Nicom | 2917 | 17.21 | 3.57 | 10.63 | 74.25 |
| *Nitrosomonas europaea ATCC 19718* | Nieur | 2287 | 53.78 | 40.58 | 53.56 | 74.64 |
| *Nocardia cerradoensis NBRC 101014* | Nocer | 6339 | 12.38 | 2.19 | 1.03 | 35.84 |
| *Nocardia otitidiscaviarum IFM 11049* | Nooti | 6593 |  | 2.64 | 2.11 | 18.35 |
| *Pseudomonas azotoformans S4* | Psazo | 5642 | 8.10 | 15.44 | 30.11 | 1.44 |
| *Pseudomonas citronellolis P3B5* | Pscit | 5583 | 49.58 | 42.20 | 68.21 | 6.05 |
| *Pseudomonas fluorescens A506* | Psflu | 4916 | 14.22 | 12.73 | 28.58 | 3.05 |
| *Pseudomonas mendocina NK-01* | Psmen | 4626 | 6.77 | 6.85 | 12.08 | 0.30 |
| *Pseudomonas oryzihabitans USDA-ARS-USMARC-56511* | Psory | 4019 | 24.16 | 2.61 | 41.48 | 6.84 |
| *Pseudomonas putida 1A00316* | Psput | 4598 | 41.13 | 44.45 | 44.89 | 0.15 |
| *Rhizobium gallicum IE4872* | Rhgal | 6170 | 0.75 | 1.41 | 12.66 | 4.49 |
| *Streptomyces avermitilis MA-4680* | Stave | 7084 | 6.56 | 3.06 | 25.65 | 1.76 |
| *Streptomyces clavuligerus ATCC 27064* | Stcla | 6819 | 7.17 | 16.82 | 9.99 | 10.15 |
| *Streptomyces hygroscopicus limoneus KCTC 1717* | Sthyg | 8388 | 18.24 | 4.77 | 27.85 | 6.78 |
| *Streptomyces noursei ATCC 11455* | Stnou | 7600 | 15.67 | 15.97 | 29.33 | 4.89 |
| *Streptomyces rubidus CGMCC 4.2026* | Strub | 7073 | 6.53 | 3.93 | 36.11 | 3.94 |
| *Streptomyces scabrisporus DSM 41855* | Stsca | 8835 | 5.65 | 14.75 | 18.17 | 3.75 |
| *Streptomyces vitaminophilus ATCC 31673* | Stvit | 5004 | 2.82 | 8.57 | 40.59 | 3.46 |
| *Thiobacillus denitrificans ATCC 25259* | Thden | 2576 | 13.98 | 1.36 | 14.44 | 14.75 |
| *Vibrio gazogenes DSM 21264* | Vigaz | 3672 | 4.77 | 1.93 | 4.82 | 43.63 |
| *Vibrio natriegens NBRC 15636* | Vinat | 4165 | 2.26 | 65.43 | 3.53 | 41.92 |
